# Nanozymes for Environmental Pollutant Monitoring and Remediation

**DOI:** 10.3390/s21020408

**Published:** 2021-01-08

**Authors:** Elicia L. S. Wong, Khuong Q. Vuong, Edith Chow

**Affiliations:** Aperture, Ryde, NSW 2112, Australia; elicia.wong@apertureteam.com (E.L.S.W.); khuong.vuong@apertureteam.com (K.Q.V.)

**Keywords:** degradation, detection, enzyme, heavy metal, nanomaterial, nanoparticle, peroxidase, pesticide, pollution, sensor

## Abstract

Nanozymes are advanced nanomaterials which mimic natural enzymes by exhibiting enzyme-like properties. As nanozymes offer better structural stability over their respective natural enzymes, they are ideal candidates for real-time and/or remote environmental pollutant monitoring and remediation. In this review, we classify nanozymes into four types depending on their enzyme-mimicking behaviour (active metal centre mimic, functional mimic, nanocomposite or 3D structural mimic) and offer mechanistic insights into the nature of their catalytic activity. Following this, we discuss the current environmental translation of nanozymes into a powerful sensing or remediation tool through inventive nano-architectural design of nanozymes and their transduction methodologies. Here, we focus on recent developments in nanozymes for the detection of heavy metal ions, pesticides and other organic pollutants, emphasising optical methods and a few electrochemical techniques. Strategies to remediate persistent organic pollutants such as pesticides, phenols, antibiotics and textile dyes are included. We conclude with a discussion on the practical deployment of these nanozymes in terms of their effectiveness, reusability, real-time in-field application, commercial production and regulatory considerations.

## 1. Introduction

### 1.1. General Introduction to Nanozymes

Nanozymes are advanced nanomaterials which possess unique physicochemical properties with the precise structural fabrication capability to mimic intrinsic biologically relevant reactions. Specifically, nanozymes mimic natural enzymes and exhibit enzyme-like properties. The enzymatic catalytic reactions are highly effective, with reactions occurring rapidly even under mild conditions, and more importantly, such reactions are also highly selective. The high efficiency and selectivity are immensely desirable properties for sensing and monitoring applications. However, natural enzymes including proteins suffer from limitations such as low thermostability and narrow pH window, which will denature the enzymes and greatly reduce and inhibit their enzymatic activities. Low thermostability also places stringent requirements on the storage, transportation and handling of natural enzymes, which can be labour- and infrastructure-intensive for the users. Susceptible denaturation adds complexity to the interpretation of sensing and monitoring outputs, which may yield a false positive/negative outcome. From this perspective, nanozymes address these limitations by offering high structural durability and stability, while maintaining the desirable catalytic activities.

By incorporating the unique physicochemical properties and enzyme-like activities, nanozymes exhibit promising applications in different fields such as the biomedical sector (in vivo diagnostics/and therapeutics) and the environmental sector (detection and remediation of inorganic and organic pollutants). The biomedical and clinical translation of nanozymes have been extensively reviewed [1,2,3,4], from their applications in immunoassays [5,6,7,8,9,10] to cancer diagnostics and therapeutics [11,12,13,14,15]. As nanozymes offer better structural stability over their respective natural enzymes, with wider physical (e.g., temperature) and chemical (e.g., pH) operational windows, they are ideal candidates for real-time and/or remote environmental monitoring and remediation. This is especially so given the challenging and unpredictable nature of the outdoor environment (compared to a more physiologically stable in vivo or in vitro environment which has a more defined and narrower operational window). In this review, the current environmental translation of nanozymes into a powerful sensing or remediation tool through inventive (i) nano-architectural design of nanozymes and (ii) transduction methodologies will be reviewed, as well as the practical deployment of these nanozymes in terms of their functionality and recyclability.

### 1.2. Types of Nanozymes

**Figure 1:**
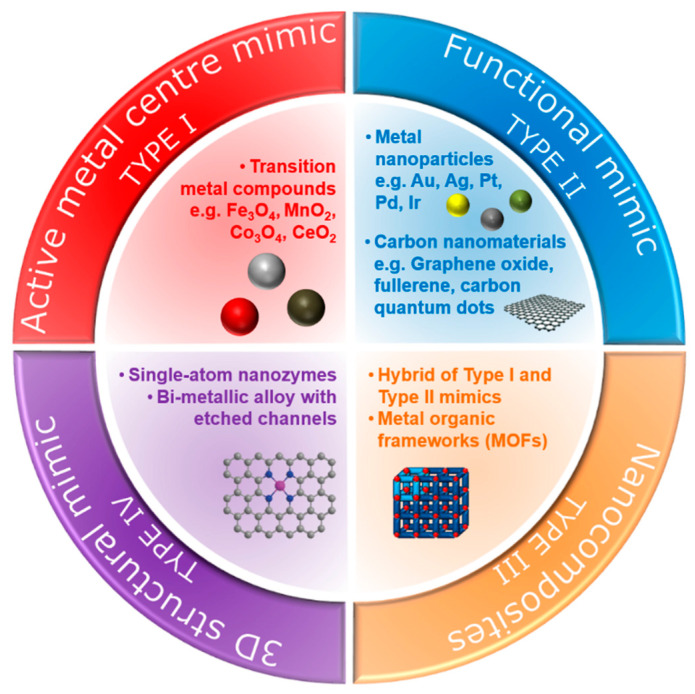
Types of nanozymes based on their mode of natural enzyme-mimicking behaviour.

Over the last few decades, various types of nanomaterials have been reported to have intrinsic enzyme-like activities [16,17]. Natural enzymes exhibit intrinsic catalytic ability, usually at a single active site, to catalyse a specific chemical transformation [18,19]. Since nanozymes lack such an active site, different strategies have been devised to enhance the catalytic properties of these nanomaterials, enabling them to selectively and effectively react with target molecules. In this review, we categorise nanozymes into four types based on their mode of natural enzyme-mimicking behaviour (Figure 1).

One strategy utilises constructed metal sites (type I nanozymes), such as metal oxides or metal sulphides, to mimic the metal catalytic active site found in metalloenzymes [20]. An early example of such nanozymes is iron oxide (Fe_3_O_4_) nanoparticles, as reported by Gao et al. [6], which exhibit peroxidase-like activity similar to the natural horseradish peroxidase (HRP) enzyme. Peroxidases catalyse the oxidation of a chromogenic substrate, such as 3,3′,5,5′-tetramethylbenzidine (TMB), 2,2′-azino-bis(3-ethylbenzothiazoline-6-sulfonic acid) (ABTS), *o*-phenylenediamine dihydrochloride (OPD), in the presence of hydrogen peroxide, H_2_O_2_. The smallest Fe_3_O_4_ nanoparticles (out of 30, 150 and 300 nm) were found to have the highest catalytic activity, which showed that the high surface area of the Fe_3_O_4_ nanoparticles was responsible for the peroxidase-like activity. Gao et al. [6] postulated that the active surface ferrous and ferric ions in the nanoparticles were the key components that enabled this catalytic activity, mimicking the iron–heme binding site in HRP. Cerium oxide (CeO_2_) nanoparticles also utilise their metal as a nanozyme due to its similarity in structure and biochemistry to the iron ion, particularly in binding to proteins [21]. In fact, CeO_2_ nanoparticles are multifunctional catalysts whereby they exhibit catalase-like (breakdown of H_2_O_2_ into O_2_ and H_2_O) and superoxidase-like (dismutation of O_2_^•−^ into O_2_ and H_2_O_2_) activities in addition to bearing peroxidase-like activity. The multifunctional catalytic behaviour arises from the coexistence of Ce(III) and Ce(IV) oxidation states. The switch between the III/IV valence resembles the mechanism of redox enzymes, which use metals as cofactors to catalyse a range of reversible redox reactions [16]. As such, reactions comprising redox cycles between Ce(III) and Ce(IV) oxidation states make it possible for CeO_2_ nanoparticles to react catalytically with oxygen radicals and hydrogen peroxide, thus mimicking the function of two key antioxidant enzymes, namely superoxide and catalase [22]. Similar to the Fe_3_O_4_ nanoparticles, the enzyme-mimicking behaviours of CeO_2_ are also size-dependent, whereby the smaller CeO_2_ (5 nm) have superior catalytic activity to that of larger (28 nm) nanoparticles [23]. This is further exemplification that the catalytic activities of the nanozymes are dependent on the amount of catalytically active atoms exposed on the surface (where the catalytic mechanism is related to changes in metal valence), which is usually inversely proportional to the diameter of the nanoparticles.

Other common metal–heme centres found in metalloenzymes include copper, cobalt and manganese ions (Table 1). As such, nanoparticles synthesised from cobalt oxide (Co_3_O_4_) [24], cobalt sulphide (Co_9_S_8_) [25], copper oxide (CuO) [26] and manganese dioxide (MnO_2_) [27,28] are also known to possess either peroxidase, oxidase and/or catalase-mimicking activities.

While the type I metal compound nanoparticles are those that mimic the metal–heme redox centre of metalloenzymes, other metal nanoparticles that catalyse the same reactions as natural enzymes are classified as type II nanozymes. These metal nanoparticles are synthesised from metals which are known to exhibit intrinsic catalytic behaviour for various heterogeneous reactions. Such metals include gold, silver, platinum, palladium and iridium [29,30,31]. Rossi and co-workers reported that gold nanoparticles, under controlled conditions (unprotected “naked” nanoparticles, 3.6 nm diameter, in the presence of excess glucose) could initially catalyse reactions similar to glucose oxidase and thus serve as a mimic for glucose oxidase [32]. Moreover, gold nanoparticles also showed peroxidase-mimicking activity [33,34]. Chen and co-workers [33] demonstrated that unmodified gold nanoparticles had significantly higher catalytic behaviours towards peroxidase substrates, which indicated that the superficial gold atoms were the key component to the observed peroxidase-like activity. Modified gold nanoparticles with different surface charges (positive or negative) also exhibited peroxidase-mimicking activity [34]. In fact, it was found that their enzyme-mimetic activities could be modulated by changing the pH of the environment (i.e., pH-switchable). Gao and co-workers [35] demonstrated that gold, silver, platinum and palladium nanomaterials exhibited peroxidase-like activities at acidic pH and catalase-like activities at basic pH [35]. The pH-switchable phenomenon was further investigated by Nie and co-workers using 1–2-nm platinum nanoparticles, which showed that the catalase-like activity was evident under basic conditions while the peroxidase-like activity was more dominant under acidic conditions [36]. The catalytic mechanism of the metal nanoparticles is different from the metal compound-based nanozymes and is related to the adsorption, activation and electron transfer of substrate (e.g., TMB, ABTS or OPD) on metal surfaces rather than a change in the metal valence of the nanomaterial.

Another intriguing aspect of metal-based nanozymes is that they can form alloys with different elemental compositions [37]. By combining the independent electronic characteristics of two metals, bimetallic nanoparticles can further exhibit unique properties through the synergetic effect of the two metals [38]. Thus, this makes it feasible to tailor the enzyme-mimicking activities by adjusting alloy compositions, classified here as type III nanozymes. In one example, He et al. showed that for Ag-M (M = Au, Pd, Pt) bimetallic alloy-based peroxidase nanozymes, the efficiency of the catalytic activity could be tuned by gradually changing the ratio of the two metals [39]. They suggested that the composition-dependent activity was from the electronic structure due to alloying. In another example, further enhancement of the multi-enzymatic activities was demonstrated by Yin, Wu and co-workers [40] using Au-Pt bimetallic nanoparticles by controlling the Pt and Au molar ratio to exhibit oxidase, peroxidase and catalase-like activities. An enhanced peroxidase-like activity of Ir-Pd nanocubes was obtained by depositing an Ir atomic layer on the surface of Pd nanocubes [41]. It was postulated that the adsorption energy of the Ir-Pd(100) surface was larger than that of the Pd(100), making it more energy-efficient to dissociate hydrogen peroxides into hydroxyl radicals.

Non-metallic nanozymes such as carbon-based nanomaterials, including fullerene and their derivatives, carbon quantum dots, carbon nanotubes and graphene oxide, are showing great promise with enzyme-mimicking capability owing to their intrinsic catalytic properties. The peroxidase, catalase and oxidase-like activities have all been reported [42,43,44,45,46,47]. In one example, Shi et al. [44] reported that carbon quantum dots exhibited peroxidase-like catalytic activity. It was concluded that the catalytic mechanism came from an increase in the electron density and mobility in the carbon quantum dots acting as effective catalytically active sites. Qu and co-workers [45] also reported that carboxyl-modified graphene oxide exhibited peroxidase-like activity, with electron transfer occurring from the top of the valence band of graphene to the lowest unoccupied molecular orbital of hydrogen peroxide. To further lower this band gap and improve the peroxidase-like behaviour, Kim et al. [46] co-doped the graphene oxide with nitrogen and boron and demonstrated a much higher catalytic behaviour than undoped graphene oxide. Besides peroxidase-like behaviour, the catalase-like behaviour was reported by Ren et al. [47] using graphene oxide quantum dots.

Metal–carbon nanocomposites have also been investigated as a strategy to further improve the catalytic activities of carbon nanozymes [48,49,50,51]. An oxidase-like nanozyme, catalysing an oxidation–reduction reaction involving oxygen as an electron acceptor, was constructed using a metal–carbon nanocomposite hybrid through doping a N-rich porous carbon with Fe nanoparticles [51]. The group suggested that the N-doped porous carbon acted as the binding sites to trap and transfer O_2_ molecules to catalytic sites and subsequently catalysed their redox reaction with the Fe nanoparticles. In another example, Guo, Zhang and co-workers [48] integrated graphene quantum dots with Fe_3_O_4_ nanoparticles and demonstrated superior peroxidase-like activities compared to individual graphene quantum dots and Fe_3_O_4_ nanoparticles. This superiority was attained from the synergistic interactions between graphene quantum dots and the Fe_3_O_4_ nanoparticles. Compared to the native HRP, this nanocomposite showed comparable, if not better, removal efficiencies for some phenolic compounds from aqueous solution, rendering it useful for industrial wastewater treatment [48].

All metal-, metal-compound- and carbon-based nanozymes rely on the high surface area, enabled either through small particle size (of the order of tens of nanometres for metal- or metal-compound-based nanozymes) or porous structure (carbon-based nanozymes) to maximise the exposure of the catalytically active atoms. Metal–organic frameworks (MOFs) which consist of metal ions as nodes and organic ligands as linkers also have highly porous structures that can be utilised as nanozymes. In this construct, the transition metal nodes containing the MOFs themselves can act as biomimetic catalysts, while the high porosity structure created by the metal–organic linkers can serve as the binding sites for the substrates. Their tuneable pore sizes, highly specific surface areas and exposed active sites provide MOFs with high catalytic efficiency [52]. In one example, Li and co-workers [53] demonstrated the use of a nanosized MOF, Fe-MIL-88NH_2_, as a peroxidase mimic. The catalytic mechanism was proposed as follows: hydrogen peroxide was adsorbed onto the surface or into the mesopores of Fe-MIL-88NH_2_, and the hydrogen peroxide was decomposed into hydroxyl radicals by iron. Other than the Fe-MOF, Cu-MOF [54], Ni-MOF [55], Pt-MOF [56] and Co/2Fe MOF [57] are also known to exhibit peroxidase-like behaviours. The bimetallic-MOF, Co/2Fe-MOF, exhibited dual enzymatic activities, peroxidase and oxidase. Additionally, Min, Chu and co-workers showed that CeO_2_-MOF acted as a hydrolase mimic (breakage of a chemical bond using water) to remove a phosphate group, PO_4_^3−^, from phosphopeptides [58]. Prussian Blue nanoparticles are an analogue of MOFs which can simultaneously behave as multienzyme mimics (peroxidase, superoxide dismutase and catalase-like activities) and were used effectively as a scavenger for reactive oxygen species [59].

Although the aforementioned metal-compound-, metal-, carbon-, nanocomposite- and MOF-based nanomaterials show promising enzyme-mimicking abilities, achieving the same level of binding affinity and specificity as natural enzymes remains a challenge. The limiting factors include (i) the density of the catalytically active surface ions (such as the metal ions) and functional groups (such as the carboxyl group in the carbon nanomaterials), and (ii) the efficiency of the catalytic mechanism. It has been demonstrated that nanozymes with a low density of active sites show much lower catalytic activities [60]. Additionally, the elemental composition and facet structure of these nanozymes cause the catalytic mechanism of nanozymes to be different and are usually more intricate than natural enzymes [37,61]. These limitations constrain the extensive applications of these standard nanozymes. Consequently, new strategies have emerged to mitigate these constraints through spatial or three-dimensional structural mimicking of the active sites of natural enzymes [62,63]. These structural mimics can be achieved by mimicking the geometry of pre-existing metal binding centres, the binding sites at the peripheral or the confined and empty space at the centre of natural enzymes (type IV nanozymes).

**Figure 2:**
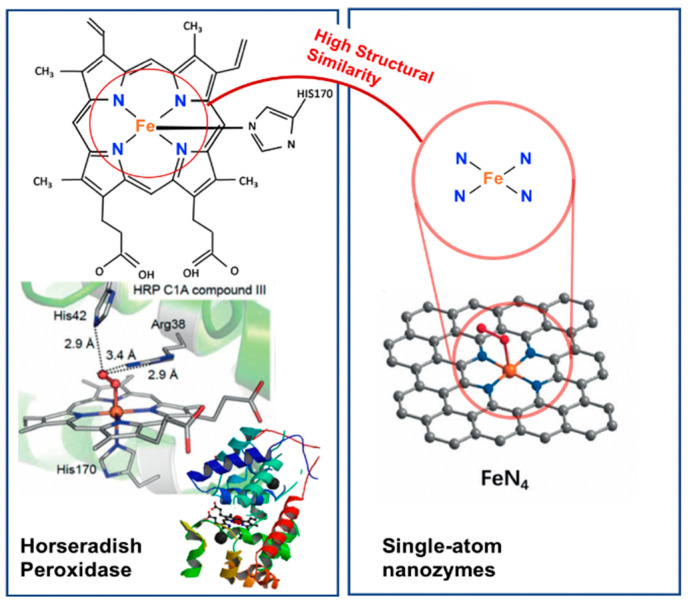
A comparison of the HRP enzyme and FeN_4_ single-atom nanozyme showing the high structural similarity of the single-atom nanozyme to the active centre (iron-heme group) of HRP. (Adapted with permission from [64]. Copyright © 2017 Wiley-VCH Verlag GmbH & Co. KGaA, Weinheim, Germany).

Single-atom nanozymes resemble spatial structures to mimic the electronic, geometric and chemical structure of the pre-existing metal binding centre of metalloenzymes. For example, the FeN_4_ in iron-based single-atom nanozymes mimic the active sites of oxymyoglobin, HRP and cytochrome P450 enzymes which contain a single heme Fe with a proximal ligand (Figure 2) [64,65]. In particular, Huang, Zhu and co-workers reported that densely isolated FeN_4_ single-atom nanozymes exhibited outstanding peroxidase-like activities [66]. Both their experimental and theoretical analyses showed that FeN_4_ led to strong adsorption of hydrogen peroxide, weakened the bonding between the single Fe atom and the two adsorbed hydroxyl groups and lowered the energy barrier for the formation of hydroxyl radicals to boost the peroxidase-like activities. Additionally, other metals such as cobalt and zinc could also be used to create CoN_4_, and ZnN_4_ single-atom nanozymes that exhibited peroxidase-like activities [66]. The addition of an axial N coordination to form FeN_5_ single-atom nanozymes enhanced the oxidase-like behaviour of the Fe based single-atom nanozymes [62]. The FeN_5_ structure had the most adsorption energy, by promoting strong oxygen adsorption that led to weakening of the O–O bond. Wang, Dong and co-workers [62] postulated that the weakening of the O–O bond was a result of the electron donor via the electron push effect of the axial-coordinated N in the FeN_5_ single-atom nanozyme.

**Figure 3:**
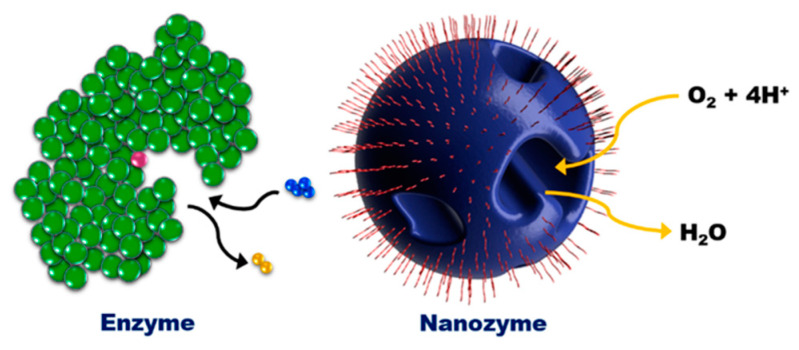
Illustration of a nanozyme as a 3D geometric architectural mimic of an enzyme. (Reprinted with permission from [63]. Copyright © 2018, American Chemical Society).

Other structure-mimicking strategies have been achieved through the creation of binding sites, such as nano-channels at the nanozymes, by resembling the binding sites of natural enzymes either at the periphery or in the centre (usually a confined empty cavity); see Figure 3. In a pioneering example, Schuhmann, Tilley, Gooding and co-workers designed a nanoparticle that mimicked the 3D architecture of a natural enzyme by using surfactant-covered PtNi bimetallic nanoparticles [63,67]. In their design, the surfactant-covered PtNi particles were selectively etched to create nano-channels that were specific for catalysing the reduction reaction of oxygen. The group reported that the oxygen reduction reaction activity normalised by the electrochemically active surface area was enhanced by a factor of 3.3 for the nanozymes compared to the unetched PtNi nanoparticles.

### 1.3. Catalytic Mechanisms

The most important advantage of nanozymes is their size-/composition-dependent activity, which enables the architectural design of nanomaterials with a broad range of catalytic activities by varying the shape, structure and composition. These nanozymes share certain similarities, such as that they need to be within a certain range of size, shape and surface charge to enable them to mimic natural enzymes. It is useful to acquire a basic insight into how these factors affect the catalytic performance of nanozymes. Thus, a fundamental understanding of the catalytic mechanisms behind the enzymatic-like activities is critical for further creative design of nanozymes with improved catalytic performance. In this section, we summarise the general mechanisms and analyse the catalytic mechanisms according to the types of nanozymes as introduced in the previous section.

**Figure 4:**
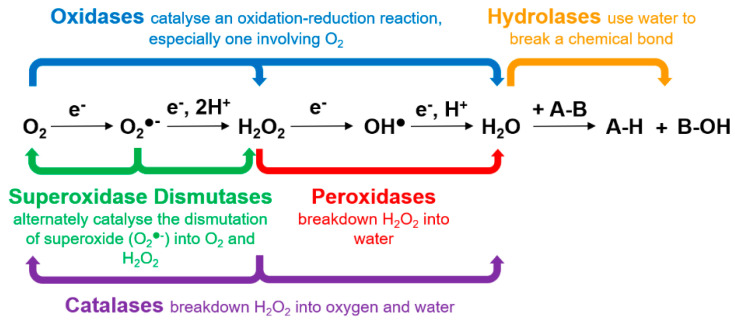
Schematic of the major reactions catalysed by oxidoreductases (oxidases, superoxide dismutases, peroxidases and catalases) and hydrolases.

Natural enzymes are internationally classified into six classes: oxidoreductases, transferases, hydrolases, lyases, isomerases and ligases. The majority of nanozymes are known to mimic the catalytic activities of oxidoreductases and hydrolases (Figure 4). Oxidoreductases catalyse oxidations and reductions in which hydrogen or oxygen atoms or electrons are transferred between molecules. Natural enzymes such as oxidases (including laccases), superoxide dismutases, peroxidases and catalases belong to the oxidoreductase family. Hydrolases catalyse the hydrolysis of various bonds and include enzymes such as phosphatase, nuclease, protease and peptidase.

The mode of catalytic reactions of natural enzymes involves two types of mechanisms, namely chemical and binding mechanisms. Chemical mechanisms include (i) acid-base catalysis (reactions involving H^+^ and OH^−^ and are pH-dependent), (ii) covalent catalysis (reactions involving formation of a transient covalent bond) and (iii) metal ion catalysis (reactions involving redox changes and stabilisation of charges). Binding mechanisms involve (iv) proximity/orientation-assisted catalysis, (v) transition state stabilisation-assisted catalysis, and vi) electrostatic catalysis. Natural enzymes use one or a combination of these actions to catalyse a chemical transformation. Understandably, nanozymes also employ one or more of these mechanisms to mimic natural enzymes.

Metal compound-based nanozymes rely on changes in metal valence to catalyse the redox reaction, and this usually involves the use of transition metal elements which have variable oxidation states. These are also the metals commonly found in the metal–heme centre of metalloenzymes as well. Metal-compound-based nanozymes include Fe_3_O_4_, Co_3_O_4_, CeO_2_, CuO and Mn_3_O_4_, in which the metal elements can be converted between variable valence states, making them promising nanozyme candidates. Using Fe_3_O_4_ nanoparticles as an example, Smirnov and co-workers [68] reported that the peroxidase-like activity of iron oxide originated mainly from the interaction of hydrogen peroxide with the ferrous ions on the surface of the nanoparticles (rather than from the dissolution of metal ions from the nanoparticles). These may follow Fenton reactions [69], which can be written as Equations (1)–(3). Other transition metals (M) follow similar Fenton reactions comprising redox cycles between M(n) and M(n + 1) oxidation states.

(1) $${\mathrm{Fe}}^{3+}+H_{2}O_{2}\overset{}{\to }{\mathrm{FeOOH}}^{2+}+H^{+}$$

(2) $${\mathrm{FeOOH}}^{2+}\overset{}{\to }{\mathrm{Fe}}^{2+}+{\mathrm{HO}}_{2}{}^{•}$$

(3) $${\mathrm{Fe}}^{2+}+H_{2}O_{2}\overset{}{\to }{\mathrm{Fe}}^{3+}+{\mathrm{OH}}^{-}+{\mathrm{HO}}^{•}$$

**Figure 5:**
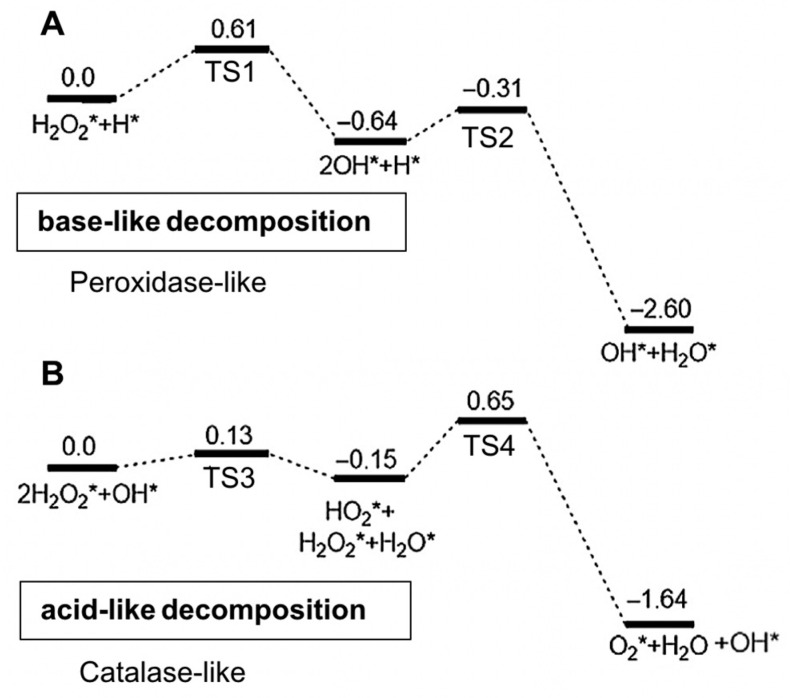
Calculated reaction energy profiles (unit: eV) for hydrogen peroxide decomposition on an Au(111) surface in (**A**) acidic and (**B**) basic conditions. Asterisk (*) is used to indicate species adsorbed on the metal surface. TS stands for transition state. (Adapted with permission from [35]. Copyright © 2015, Elsevier Ltd. All rights reserved).

The catalytic mechanism of metal nanoparticles differs from metal-compound-based nanozymes and is related to the adsorption, activation and electron transfer of the substrate (e.g., TMB, ABTS or OPD) on metal surfaces. As mentioned in the previous section, metal-based nanozymes exhibit intrinsic pH-switchable peroxidase and catalase-like activities [35]. The pH-switchable ability arises from the acid-base type of catalysis of the metal-based nanozymes, which consists of the adsorption and decomposition of hydrogen peroxide under different pH conditions. Adsorption of hydrogen peroxide first occurs on the metal surface to initiate the catalytic reaction; then, the adsorbed hydrogen peroxide undergoes two different decomposition pathways depending on the pH of the micro-environment [35]. Under acidic conditions, hydrogen peroxide follows a base-like decomposition pathway to exhibit peroxidase-like activity, whereas under basic conditions, it follows an acid-like decomposition pathway exhibiting catalase-like activity. Figure 5 illustrates the decomposition mechanisms under acidic and basic conditions. Similar to the peroxidase and catalase-like activities, the catalytic mechanism for oxidase-like behaviour was demonstrated by Wu, Gao and co-workers [37] using density functional theory to involve the adsorption of oxygen to the metal surface, followed by the dissociation of oxygen. The proposed mechanism for the oxidase-like activity is shown in Equations (4) and (5). The same catalysis mechanisms as shown in Figure 5 and Equations (4) and (5) were also applicable to bimetallic [35,37,40,70,71] and MOF-based [72] nanozymes.

(4) $$O_{2}=2O^{*}$$

(5) $$O^{*}+S\overset{}{\to }H_{2}O^{*}+S_{\mathrm{ox}}$$

* is used to indicate species adsorbed on metal surfaces. S is a chromogenic substrate; S_ox_ is the oxidised chromogenic substrate.

**Figure 6:**
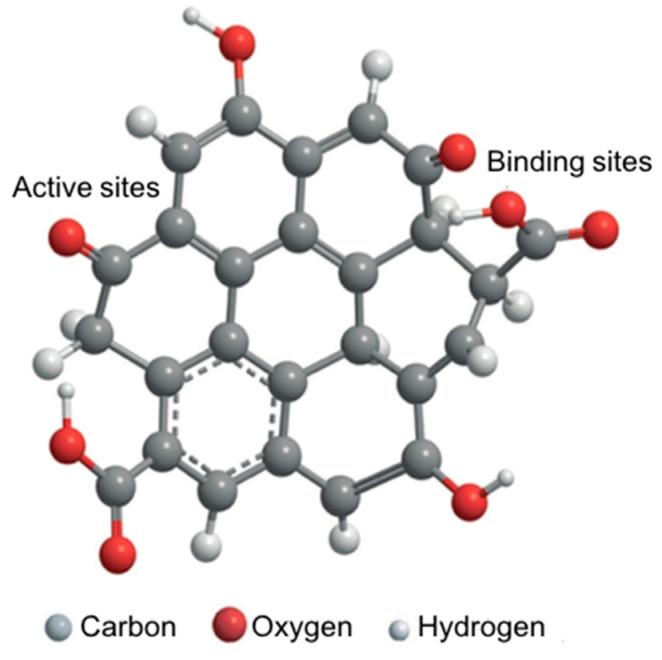
The catalytically active sites (for hydrogen peroxide) and binding sites (for the chromogenic substrate) of graphene oxide quantum dots which exhibit peroxidase-like activity. (Adapted with permission from [73]. Copyright © 2015 WILEY-VCH Verlag GmbH & Co. KGaA, Weinheim, Germany).

Unlike metal-compound- and metal-based nanozymes, the catalytic mechanism of carbon-based nanozymes is not as well documented. However, it follows a catalytic route as for the metal-based nanozymes involving the adsorption, activation and electron transfer of the substrate at carbon surfaces. Qu and co-workers [43,73] completed a comprehensive study of the mechanism of the peroxidase-like activity of graphene oxide quantum dots, which stemmed from their ability to catalyse the decomposition of hydrogen peroxide and generate highly reactive hydroxyl radicals. These catalytic reactions occur at two sites, reactive and substrate-binding sites: the functional groups –C=O act as the catalytically active sites for converting hydrogen peroxide to hydroxyl radicals, and the O=C–O– groups serve as the substrate-binding sites for hydrogen peroxide (Figure 6). In a separate study, Zhao et al. [74] also reported a similar catalytic mechanism where the hydrogen peroxide attacked the –C=O group and H_2_O_2_ decomposed to form a hydroxyl radical.

For nanozymes that rely on structural analogy with the natural enzyme as the strategy to achieve the desired enzyme-mimicking properties, the high structural similarity maximises the binding energy of the substrate and lowers the activation energy required to initiate the catalytic reaction. Single-atom dispersion (for single-atom nanozymes) also generates the most sufficient size effect and provides maximum surface-active site exposure [75] because the catalytic mechanism depends primarily on the steric configuration of active centres instead of the size or structure of the nanoparticles as seen in the metal- and metal-compound-based nanozymes [62]. Using single-atom nanozymes with carbon nanoframe-confined FeN_5_ active centres, Wang, Dong and co-workers [62] reported both experimental and theoretical studies of the catalytic mechanism of their oxidase-like activity. The sterically configured metal active centres (FeN_5_) have a strong O_2_ adsorption to the surface, weakening the O–O bond and giving rise to a larger extent of O–O bond elongation, thereby promoting the oxidase-like activity. Additionally, Xu et al. [76] also reported a similar catalytic mechanism for the peroxidase-like activity of single-atom nanozymes with the adsorption of hydrogen peroxide to the sterically configured active centres and the decomposition of hydrogen peroxide into reactive radicals.

Instead of using the single-atom dispersion approach to maximise the surface-active site exposure, Calle-Vallejo et al. [77] introduced the concept of “coordination–activity plots” to predict the geometric structure of the optimal active sites. This involved counting the number of neighbouring atoms to the reaction active site rather than using the more conventional Sabatier principle to predict the activity of the catalyst based on the strength of the adsorption and desorption of key intermediates. Benedetti et al. [63] utilised the “coordination–activity plot” to design their nanozymes to structurally mimic the natural enzyme using a bimetallic alloy with etched nano-channels and demonstrated an improvement in the electrocatalytic performance of such a nanozyme.

In the following sections, the environmental applications of nanozymes will be discussed in greater detail. In particular, the administration of nanozymes for pollutant detection and degradation are intricate functions of the physicochemical properties of different types of nanozymes. Therein, (i) the ability to structurally manipulate the nanoparticles with atomic precision which affords specificity towards the detection of targeted pollutants, (ii) the surface engineering, such as the coatings applied, to improve the sensitivity of the detection and efficiency of biodegradation, and (iii) the transduction methods applied to enable the detection and biodegradation of pollutants are reviewed.

## 2. Detection of Metal Ions

### 2.1. Introduction

The persistence of toxic heavy metals in the environment is an important concern that can lead to adverse effects on human health. Heavy metals occur naturally in water, food and soil but also arise from industrial activities such as mining, smelting, electroplating, wood preserving and leather tanning [78]. In comparison to organic contaminants, inorganic ions are not biodegradable and tend to accumulate in the environment and organisms and enter the food supply chain. Effects from heavy metal exposure include nausea, vomiting, diarrhoea, abdominal pain, kidney dysfunction and, in extreme cases, death [78]. Therefore, there is a critical need for highly sensitive and selective sensors for monitoring metal ions. The Australian Drinking Water Guidelines for selected metals are listed in Table 2 [78].

Analytical instrumentation such as atomic absorption spectroscopy (AAS), inductively coupled plasma–optical emission spectroscopy (ICP-OES) and inductively coupled plasma–mass spectrometry (ICP-MS) [79] can address the needs of highly accurate and sensitive metal ion detection but require sample processing and analysis back in the laboratory as well as operation by trained personnel. Due to the high cost of this instrumentation, it are not easily accessible. Even where access to analytical laboratory testing facilities is available, the cost for analysis of a single sample is of the order of AUD 50–100 (USD 35–70) with a turnaround time of 2–5 days. This inherently limits the frequency of sampling as well as the number of collection points, which, in some instances, reduces the ability to rapidly identify the pollutant and its source.

**Table 2:** Australian Drinking Water Guidelines for Selected Metals [78].

Metal	Guideline Value (mg/L)	Potential Source
Arsenic	0.01	From natural sources and mining/industrial/agricultural wastes.
Cadmium	0.002	Indicates industrial or agricultural contamination; from impurities in galvanised (zinc) fittings, solders and brasses.
Chromium (VI)	0.05	From industrial/agricultural contamination of raw water or corrosion of materials in distribution system/plumbing.
Copper	2	From corrosion of pipes/fittings by salt, low-pH water.
Lead	0.01	Occurs in water via dissolution from natural sources or household plumbing containing lead (e.g., pipes, solder)
Mercury	0.001	From industrial emissions/spills. Very low concentrations occur naturally.
Silver	0.1	Concentrations are generally very low. Silver and silver salts occasionally used for disinfection.

Ideally, sensors which are portable and amenable for in-field sensing can reduce analysis times and provide real-time information as to the health state of the environment. Solid-state sensors that rely on colorimetric or electrochemical transduction can address these challenges as they are more compact and simpler to operate than lab-based analytical instrumentation. These sensors will ultimately lead to cost savings, as timely reporting allows rectifying actions to be taken before conditions worsen. Moreover, humans, animals and aquatic life will be prolonged by reducing the risk of exposure to these toxic elements.

Most colorimetric [80,81] or electrochemical transduction techniques [82,83] rely on a chemically sensitive material to interact with the metal ion of interest via coordination, adsorption or precipitation to induce a change in output signal. Detection of metal ions using metal nanoparticles generally involves the aggregation of nanoparticles to induce a colour change [84]. The aggregation of nanoparticles is facilitated by surface ligands which bind to the metal ion of interest and crosslink the nanoparticles, resulting in a shift in the surface plasmon resonance. Ionophores and porphyrins are also ligands that have been used to coordinate metal ions with high selectivity [85] and can be tethered directly to a substrate or immobilised within a polymer matrix. Not surprisingly, oligopeptides, DNA and enzymes [82,86,87,88] have also been studied for metal chelation since, in nature, metal ions play a key role in their interaction with living organisms. Metal ions can be highly toxic to enzymes since the thiol or methylthiol groups of amino acids near the active centre of enzymes can be blocked by heavy metals and inhibit their function [89].

In the past decade, several research groups have explored the use of nanozymes for the detection of metal ions [90,91,92,93]. Similar to natural enzymes, the peroxidase, oxidase or catalase-like activity of nanozymes can be inhibited or enhanced by the presence of metal ions. Many studies exploit the intrinsic peroxidase-like activity of nanozymes and are mainly type II metal nanoparticles [94,95,96,97,98,99,100,101]. These metal nanoparticles form an amalgam upon interaction with a metal ion or agglomerate through binding of the metal ion with the surface ligands of nanoparticles, changing the enzyme-like activity. The peroxidase-like activity of type I nanozymes such as cysteine-stabilised Fe_3_O_4_ magnetic nanoparticles (MNPs) [102], chitosan-functionalised MoSe_2_ [103] and Co_9_S_8_ [104] can also be triggered by metal ions. Nanocomposite type III nanozymes have also been explored through the synergistic combination of metal nanoparticles, graphene-based materials or MOFs [56,105,106,107,108,109]. These nanocomposites can serve to enhance the catalytic activity or are dual-purpose nanozymes for the adsorption and detection of the metal ion of interest. Studies exploiting the oxidase [110,111] or catalase-like [112,113] activity of nanozymes for metal ion detection are limited. Whilst most peroxidase-like nanozymes exhibit high metal ion selectivity, the demonstrated examples of catalase-like nanozymes were cross-selective [112,113] with the ability to detect multiple metal ions under various conditions. The proceeding sections will review the various metal ions and their detection using nanozymes.

### 2.2. Mercury Detection

Mercury is a highly toxic heavy metal which naturally occurs in water, soil and even food. Due to its wide range of adverse effects on human health, including tremors, mental disturbances and gingivitis [78], many efforts have been focused on developing highly sensitive and selective Hg^2+^ sensors.

**Figure 7:**
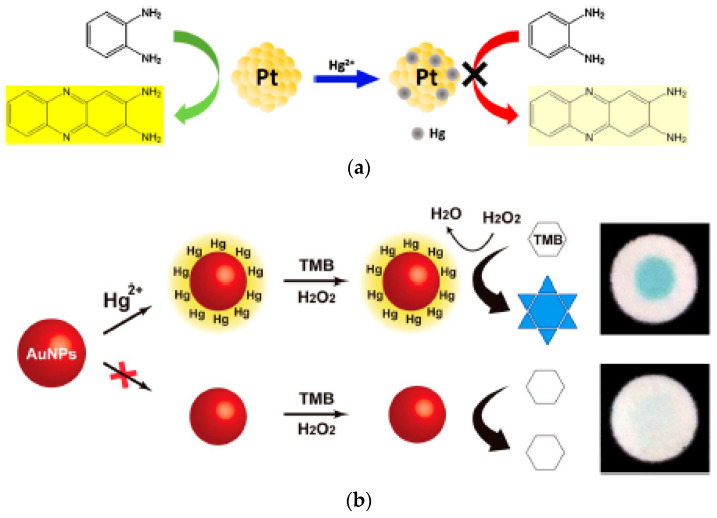
(**a**) Detection of Hg^2+^ ions by inhibition of OPD activity by formation of a Pt-Hg amalgam. (Reprinted with permission from [96]. Copyright © 2017, Elsevier B.V. All rights reserved.) (**b**) Detection of Hg^2+^ by enhancement of TMB activity by formation of an Au-Hg amalgam. (Reprinted from [114]. Copyright © The Authors. Distributed under a Creative Commons BY (CC BY) license).

Long et al. [101] first reported a colorimetric sensor for Hg^2+^ based on the peroxidase-like enhancement of gold nanoparticles. TMB, a chromogenic substrate, can be oxidised by H_2_O_2_ in the presence of citrate-capped gold nanoparticles, resulting in a visual colour change from colourless to light blue. Remarkably, the addition of trace Hg^2+^ to the citrate-capped gold nanoparticles dramatically enhanced the oxidation of TMB, with the colour intensity proportional to the Hg^2+^ concentration. Compared to other metal ions at 25 times higher concentration, including K^+^, Na^+^, NH_4_^+^, Ag^+^, Ba^2+^, Mg^2+^, Co^2+^, Ni^2+^, Zn^2+^, Cu^2+^, Mn^2+^, Pb^2+^, Cd^2+^, Al^3+^, Cr^3+^ and Fe^3+^, the extent of oxidation of TMB was unaltered. The enhancement of peroxidase-like activity due to Hg^2+^ was attributed to two processes. In the first step, Hg^2+^ was reduced to Hg^0^ by sodium citrate, which also acted as a stabilising agent for the nanoparticles. Subsequently, the reduced mercury dispersed across the gold nanoparticle surface, forming a Hg-Au amalgam, and improved the peroxidase-like activity of the nanoparticles. The altered nanoparticle surface enhanced the formation of ^●^OH radicals on the surface due to bond breakage of H_2_O_2_. Similar phenomena were also observed with MoS_2_-Au [108], Cu@Au nanoparticles [94] and Au/Fe_3_O_4_/graphene oxide [109]. In the latter case, the catalytic activity of Au/Fe_3_O_4_/graphene oxide was improved in the presence of Hg^2+^, resulting in ultrasensitive detection of Hg^2+^ down to 0.15 nM [109]. Furthermore, mercury was able to be removed (>99% efficiency) from the surface of Au/Fe_3_O_4_/graphene oxide by the application of an external magnetic field, allowing reuse up to 15 times. In an approach towards rapid, on-site testing [114], Han et al. have developed a paper-based analytical device for Hg^2+^ detection based on citrate-stabilised gold nanoparticles (see Figure 7a). The device was designed so that multiple samples could be analysed simultaneously by having separate zones for reagent loading, absorbents and detection. By varying the number of drops of the test sample, colorimetric signal amplification due to the catalytic reaction of TMB and H_2_O_2_ could be realised.

Whilst the peroxidase-like activity of gold nanoparticles was stimulated by Hg^2+^, the application of platinum nanoparticles resulted in enzyme inhibition. Similar to gold, the mechanism for a change in catalytic activity of platinum-based nanoparticles was a result of Hg-Pt amalgam formation at the Pt surface, affecting the electronic structure and active Pt^0^ proportion. However, Hg^2+^ can interact directly with Pt^0^ via metallophilic interactions due to their matched d^10^ configuration, resulting in catalytic inhibition [91]. Li et al. [95] were the first to demonstrate in 2015 that the peroxidase-like activity of Pt nanoparticles stabilised by bovine serum albumin (BSA) could be inhibited through interactions between Hg^2+^ and Pt^0^, allowing the detection of Hg^2+^ down to 7.2 nM in 20 min without significant interference from other metal ions. Similarly, Zhou et al. [96] have used citrate-stabilised Pt nanoparticles for the detection of Hg^2+^ ions. The OPD colorimetric signal was inhibited through the reduction of Hg^2+^ by citrate, resulting in a Pt-Hg amalgam (see Figure 7b). To enable screening in-field in various water bodies and public drinking water distribution systems, Kora and Rastogi [115] have developed a green approach for the synthesis of platinum nanoparticles using chloroplatinic acid and a nontoxic, biodegradable plant exudate gum as a reducing and stabilising agent. These nanoparticles (with an average size of 4.4 nm) showed excellent tolerance to temperature and pH changes and exhibited peroxidase-like activity.

Although the majority of Pt-based nanomaterials exhibit peroxidase-like activity, the oxidase-like activity has also been demonstrated, which is more ideal for environmental detection since unstable H_2_O_2_ is not required. Doping of Pt nanostructures with Se showed oxidase-like activity [110] due to an acceleration of electron transport and the anchoring of Se to Pt nanoparticles. The presence of Hg^2+^ inhibited the catalytic activity, enabling a facile colorimetric assay for Hg^2+^ down to 70 nM.

**Figure 8:**
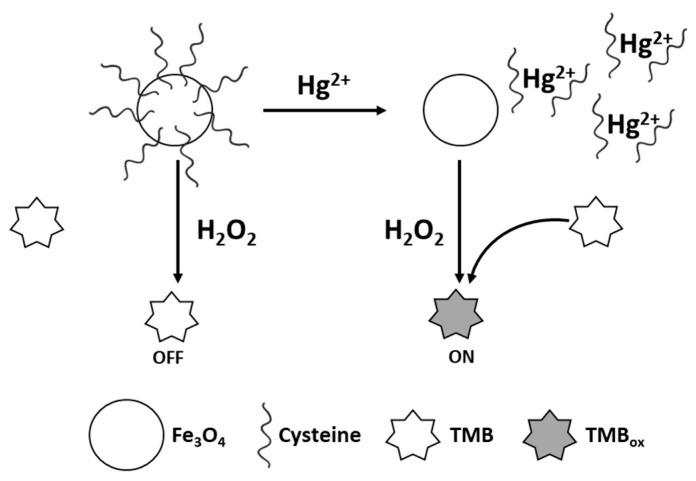
Principle for sensing of Hg^2+^ based on Hg^2+^-triggered peroxidase-mimicking activity of Cys-Fe_3_O_4_ nanoparticles. (Adapted with permission from [102]. Copyright © 2018, Elsevier B.V. All rights reserved.).

The enzyme-mimicking properties of nanomaterials can also be enhanced or inhibited through surface modification. Iron oxide nanoparticles are known to exhibit peroxidase-like activity, but their activity can be inhibited through blockage of the active Fe sites [6]. Cysteine-modified Fe_3_O_4_ nanoparticles [102] exhibited almost no colour change in the presence of TMB and H_2_O_2_ under pH 4.0 conditions. However, the presence of Hg^2+^ triggered the peroxidase-like activity of Fe_3_O_4_ due to the formation of a cysteine–Hg^2+^–cysteine complex (Figure 8). Excellent sensitivity was achieved, with a detection limit of 5.9 nM. Detection of Hg^2+^ was also enhanced using gold nanoparticles modified with chitosan [97]. Hg^2+^ can react with chitosan via the NH_2_ groups. By using TMB as a chromogenic substrate, and with 10-min incubation at 50 °C in pH 4.2 acetate buffer, the absorption peak at 652 nm was dramatically improved due to Hg^2+^. Selenium nanoparticles have also been shown to catalyse the oxidation of TMB by surface modification with chitosan. Compared to BSA and sodium alginate Se nanoparticles, the chitosan Se nanoparticles had the highest activity, which was attributed to the involvement of reactive oxygen species that may react more favourably with chitosan [111]. The oxidase-like activity of chitosan-stabilised Se nanoparticles could be inhibited by Hg^2+^ due to the extremely high affinity of mercury for selenium. High selectivity for Hg^2+^ (5 μM) was afforded when evaluated under the same conditions as Cd^2+^, Pb^2+^, Cu^2+^, Na^+^, K^+^, Ca^2+^, Mg^2+^, Zn^2+^, Mn^2+^, Al^3+^ and Fe^3+^ (100 μM). Similarly, Huang et al. [103] have demonstrated that chitosan-functionalised molybdenum (IV) selenide exhibited peroxidase and oxidase-like activities. The chitosan surface functionality enabled the reduction of Hg^2+^ ions to Hg^0^ and further enhanced the enzyme activity. The selectivity of the nanozyme to Hg^2+^ (2 μM) was well-demonstrated over other ions at 10 μM.

The use of nanocomposites has also been gaining popularity as enzyme mimics. Combining metal nanoparticles with support materials such as graphene and MOFs is highly attractive as they may result in higher activities owing to synergistic interactions [56,105,108,109]. Gold nanoparticles dispersed onto a porous amino-functionalised titanium-based MOF exhibited peroxidase-like behaviour [105] that was higher than the individual gold nanoparticles and titanium-based MOF. Furthermore, kinetic analysis of the nanocomposite revealed a lower Michaelis constant value, *K*_m_ (i.e., a higher affinity for the substrate), than HRP. The presence of cysteine inhibited the peroxidase-like behaviour but could be reactivated by Hg^2+^ due to the strong affinity of Hg^2+^ for the thiol group of cysteine. The detection of Hg^2+^ was interfered with by Cu^2+^ and Ag^+^ but could be partially masked with ethylenediaminetetraacetic acid (EDTA) and Cl^−^. MOFs have also been decorated with Pt nanoparticles [56]. Combining the high peroxidase-like activity of Pt nanoparticles with the high porosity and surface area of MOFs provided a rapid technique for the measurement and removal of Hg^2+^ ions. Hg^2+^ ions inhibited the peroxidase-like activity, with high sensitivity and a Hg^2+^ detection limit of 0.35 nM, without significant interference from coexisting metal ions. Furthermore, by using 5 mg of the nanocomposite, effective removal (>99%) of Hg^2+^ (up to 50 mg/L) could be demonstrated after 12-h incubation. It is envisaged that advances in nanomaterials would result in the rise of more dual-purpose materials that can be used for both sensing and adsorption of metal ions.

### 2.3. Lead Detection

**Figure 9:**
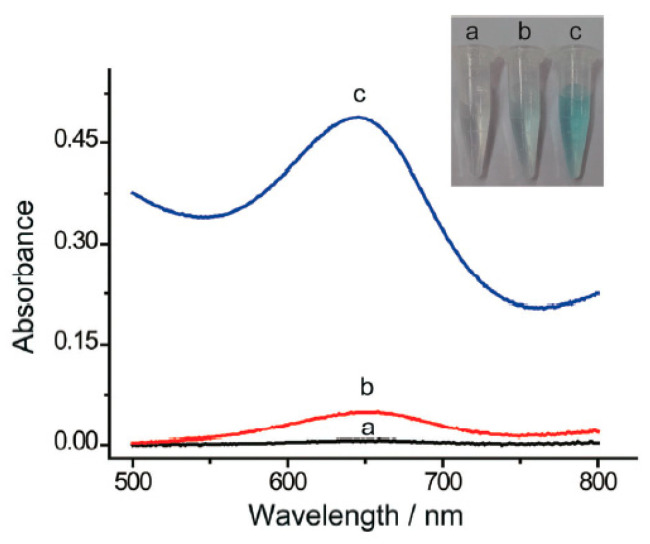
Absorption spectra and image (inset) of 500 μM TMB in the presence of (**a**) 10 mM H_2_O_2_, (**b**) 10 mM H_2_O_2_ and 5 μg/mL^−1^ Au nanoclusters, and (**c**) 10 mM H_2_O_2_, 5 μg/mL^−1^ Au nanoclusters and 250 μM Pb^2+^. (Reprinted with permission from [98]. Copyright © 2017, Royal Society of Chemistry.).

Lead is a cumulative poison that can have a major detrimental effect on the central nervous system [78]. Considering the potential health effects, the permissible Pb(II) level for Australian drinking water is set at 10 μg/L [78]. Thus, it is imperative to provide simple methods for monitoring Pb^2+^ in a variety of samples (environmental and food). Natural enzymes, such as DNAzymes [116], have high catalytic activity for Pb^2+^ and have been employed as biosensors for Pb^2+^. In recent years, enzyme-mimicking nanomaterials for the detection of lead ions have gained popularity, involving enhancement and inhibition strategies. Gold nanoclusters modified with glutathione exhibit weak peroxidase-like activity [98] but the presence of Pb^2+^ ions can induce the aggregation of the gold nanoclusters due to binding with glutathione (Figure 9). The catalytic activity of TMB could be increased ten-fold upon aggregation, with a Pb^2+^ detection limit of 2 μM. Other bivalent metal ions (Ca^2+^, Cd^2+^ and Zn^2+^) capable of forming complexes with glutathione interfered with the detection of Pb^2+^, but at much higher concentrations. Au@Pt nanoparticles [117] have also been used for the determination of Pb^2+^ ions through inhibition of the peroxidase-like activity. In the presence of sodium thiosulfate, Pb^2+^ ions accelerated the leaching of gold, which induced slight aggregation of the gold nanoparticles from a hydrate size of 35.5 to 75.0 nm. The aggregated nanoparticles exhibited weakened peroxide activity and could be correlated to Pb^2+^ concentration without interference from other metal ions. A detection limit of 3.0 nM Pb^2+^ was achieved. In another example, layered WS_2_ nanosheets [118] also showed peroxidase-like activity which could be inhibited by Pb^2+^ ions. The high selectivity towards Pb^2+^ was attributed to the layered structure of the nanomaterial, which had a stronger adsorption capacity for Pb^2+^ than other metal ions. The nanozyme also offered high sensitivity (detection limit of 4 μg/L), which provided an easy method to distinguish whether the permissible Pb^2+^ level was exceeded even with the naked eye.

### 2.4. Silver Detection

**Figure 10:**
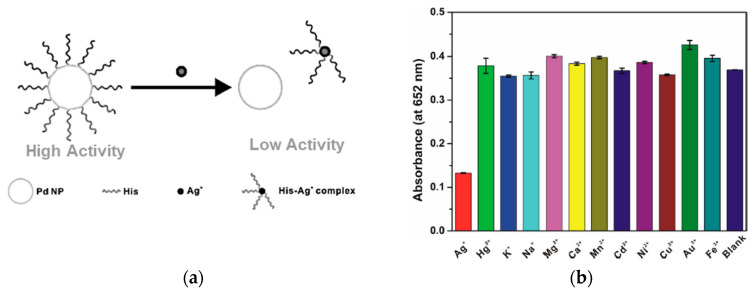
(**a**) Mechanism for the peroxidase-like activity of histidine-modified Pd nanoparticles and its effect in the presence of Ag^+^. (**b**) Selectivity of the Pd nanoparticles for Ag^+^ compared to other metal ions as demonstrated by a decrease in oxidised TMB colour formation. (Reprinted with permission from [99]. Copyright © 2018, Elsevier B.V. All rights reserved).

Silver is widely used in the electrical, photography and pharmaceutical industries and has resulted in increased levels of silver in the environment. Bioaccumulation of silver in the human body can lead to argyria, which results in bluish-grey metallic discolouration of the skin, hair, mucous membranes, mouth and eye [78]. Thus, it is vital to monitor the presence of silver in environmental and biological samples. Nanozymes for Ag^+^ detection based on catalytic enhancement [106] and inhibition [99,100] have both been demonstrated. To more closely mimic natural enzymes, Zhang et al. [99] have modified Pd nanozymes with histidine. Amino acids are attractive surface modifiers for nanoparticles due to their biocompatibility and metal chelation ability. Histidine-modified Pd nanoparticles prepared by using histidine as a stabiliser and NaBH_4_ as a reducing agent offered enhanced TMB colour formation (peroxidase-like activity) compared to bare Pd particles, which were formed in the absence of histidine. In the presence of Ag^+^, specific binding to histidine resulted in the Pd nanoparticles being exposed, and the peroxidase-like activity was suppressed. A limit of detection down to 4.7 nM was achievable. No significant suppression of the TMB colour was observed in the presence of Hg^2+^, K^+^, Na^+^, Mg^2+^, Ca^2+^, Mn^2+^, Cd^2+^, Ni^2+^, Cu^2+^, Au^3+^ or Fe^3+^ ions at the same concentration as Ag^+^ (Figure 10). In another example of peroxidase-like inhibition, Au clusters stabilised by BSA were developed by Chang et al. [100]. The introduction of Ag^+^ as low as 0.204 μM selectively reacted with Au^0^ through redox reaction, which suppressed the oxidation of TMB by the BSA-stabilised gold clusters.

In order to shift away from the high cost of noble metal nanozymes, Li et al. [106] have developed zeolitic imidazolate frameworks-9/graphene oxide (ZIL-8-GO) as a peroxidase-mimicking nanozyme. The introduction of Ag^+^ greatly enhanced the peroxidase-like activity and there was no significant interference from other metal ions even at 250 μM. Detection of Ag^+^ was achieved visually by spotting filter paper with the reagents, offering the practicality of testing real samples. The Ag^+^ detection limit using the naked eye was 0.1 μM, compared to 1.43 nM using UV–Vis spectrophotometry in a sample cuvette.

### 2.5. Copper Detection

Constant monitoring of Cu^2+^ in environmental and drinking water is necessary since an intake of excess Cu^2+^ can cause liver or kidney diseases [78]. Many colorimetric assays have been developed for Cu^2+^ sensing [119,120] but the use of nanozymes has been limited. Recently, urchin-like Co_9_S_8_ nanomaterials [104] with needle-like nanorods were found to be intrinsically catalytic due to their ability to facilitate electron transport as a variable-valence metal sulphide between TMB and H_2_O_2_. The addition of cysteine switched the Co_9_S_8_ sensor to its “off” state due to TMB radical restoration by the thiol functionality of cysteine. However, the presence of Cu^2+^ reactivated the sensor back to its “on” state, with the ability to achieve excellent selectivity and a Cu^2+^ detection limit of 0.09 μM. Glutathione can also influence the catalytic activity of nanozymes such as Pt nanoparticles and play an important role in Cu^2+^ detection [121]. Pt nanoparticles exhibit oxidase-like activity as they can effectively catalyse the oxidation of TMB by O_2_. Glutathione inhibited the oxidase-like activity but its activity could be regained in the presence of Cu^2+^ due to oxidation of glutathione. Cu^2+^ detection was demonstrated in human serum but could also potentially be applied to environmental systems.

### 2.6. Chromium Detection

Elevated levels of chromium in the environment exist due to industrial activities such as the prevention of corrosion on metal surfaces [122]. Cr(VI) is highly toxic and can easily spread and bioaccumulate. Wang et al. [107] have developed an oxidase-like nanozyme that can detect Cr(VI) over a range of 0.03–5 μM with high selectivity. The presence of Cr(VI) and cerium oxide nanorod-templated MOFs boosted the oxidation of TMB substrate. The applicability of the nanozyme was demonstrated in spiked water samples, illustrating the potential for trace metal analysis in environmental waters.

### 2.7. Arsenic Detection

Arsenic poisoning stemming from long-term exposure to contaminated drinking water is a serious concern. In some parts of Australia [78], and in countries such as Argentina, Bangladesh, Chile, China, India, Mexico and the United States of America, concentrations of naturally occurring arsenic may exceed safe levels [123]. Despite the importance of arsenic detection, there are only a few examples of nanozymes being used for arsenic detection, all of which were published recently [124,125]. Cobalt oxyhydroxide (CoOOH) nanoflakes exhibit peroxidase-like activity [125] and can bind specifically to arsenate, As(V), via electrostatic attraction and As-O interactions. As(V) inhibited the oxidation of ABTS, resulting in a colorimetric detection limit of 3.72 ppb. By exploiting the redox conversion between ABTS and ABTSox, electrochemical detection of arsenate via chronoamperometry at a CoOOH-modified glassy carbon electrode resulted in even higher sensitivity, with a detection limit of 56.1 ng/L. Similarly, Zhong et al. [124] demonstrated an electrochemical and optical method for the determination of As(V) by using the peroxidase-like activity of iron oxyhydroxide (FeOOH) nanorods. The presence of As(V), as low as 0.1 ppb, inhibited the ability of the nanorods to catalyse the oxidation of ABTS substrate by hydrogen peroxide.

### 2.8. Detection of Multiple Metal Ions

**Figure 11:**
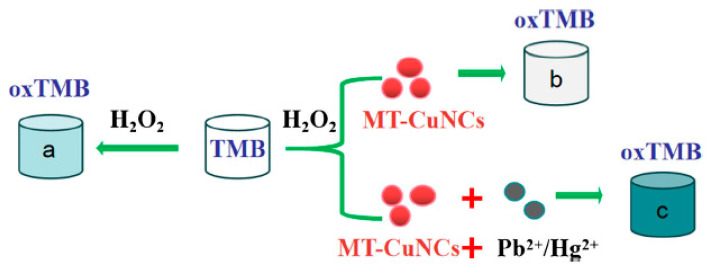
Oxidation of TMB by H_2_O_2_ (product a), catalase-like activity of metallothionein-stabilised copper nanoclusters (product b) and peroxidase-like activity of metallothionein-stabilised copper nanoclusters in the presence of Pb^2+^/Hg^2+^ (product c). (Reprinted with permission from [113]. Copyright © 2019, Springer-Verlag GmbH Austria, part of Springer Nature).

Most of the reported nanozymes exhibited high selectivity, which is ideal for monitoring a specific metal ion. However, due to the coexistence of multiple metal ions in environmental waters, a simple test that can monitor a suite of metal ions is highly desirable. Depending on the nature of the nanozyme, some were sensitive to multiple metal ions. Metallothionein-stabilised copper nanoclusters displayed catalase-like activity [113] due to their ability to decompose H_2_O_2_ and inhibit the oxidation of TMB. Interestingly, Pb^2+^ and Hg^2+^ ions were able to induce the conversion of the catalase-like activity to peroxidase-like activity (Figure 11), with detection limits down to 142 and 43.8 nM, respectively. The assay displayed potential for simultaneously monitoring toxic Pb^2+^ and Hg^2+^ in environmental water samples.

**Figure 12:**
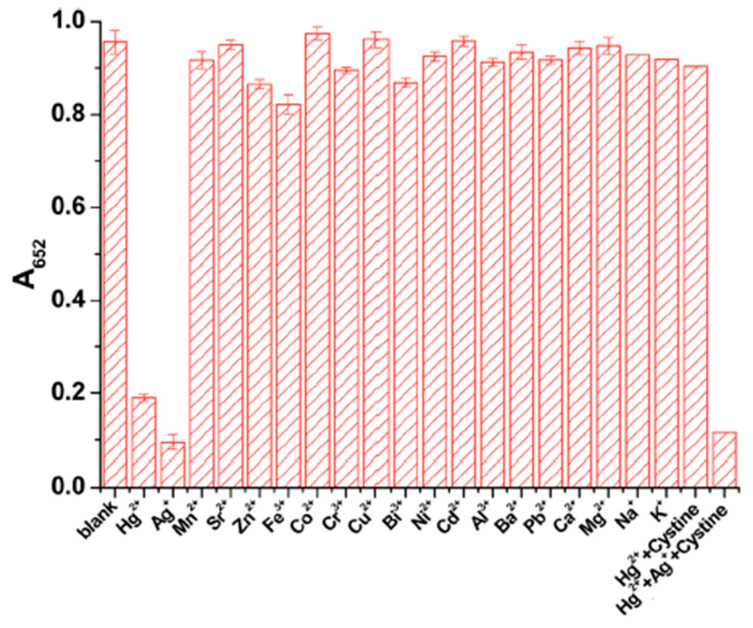
Selectivity of Au@Pt nanoparticles towards various metal ions in the presence of sodium dodecyl sulfate. (Reprinted with permission from [126]. Copyright © 2017, Royal Society of Chemistry).

Mercury and silver usually coexist in water, and peroxidase-like nanozymes have been developed that are sensitive to both metal ions. Surface modification of Au@Pt nanoparticles with sodium dodecyl sulfate effectively shielded most metal ions via complexation, except for Hg^2+^ and Ag^+^ [126]. The detection limits for Hg^2+^ and Ag^+^ were 3.5 and 2.0 nM, respectively. To discriminate Hg^2+^ and Ag^+^, cystine was added to shield Hg^2+^ as a result of cystine–Hg^2+^ binding interaction (Figure 12). In a similar approach, Zhao et al. [127] have used EDTA to mask Hg^2+^, providing a sensitive and selective means to detect both Hg^2+^ and Ag^+^ using the peroxidase-mimicking ability of polyvinylpyrrolidone-coated platinum nanoparticles.

**Figure 13:**
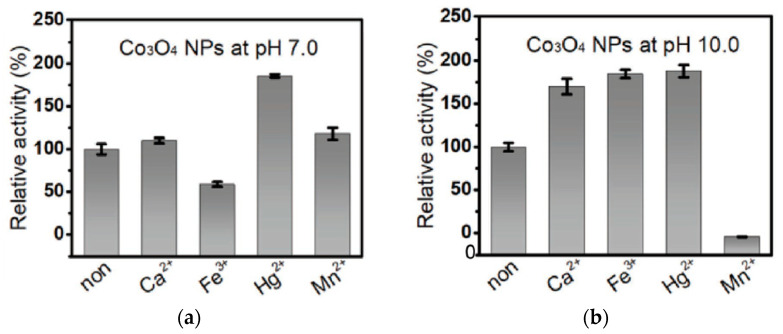
Influence of metal ions on the catalase-like activity of Co_3_O_4_ nanoparticles at (**a**) pH 7.0 and (**b**) pH 10.0. (Reprinted with permission from [112]. Copyright © 2018, John Wiley and Sons).

Han et al. [112] have exploited the pH-sensitive catalase-like activity of Co_3_O_4_ MNPs to discriminate metal ions. The nanoparticles were insensitive to pH in the range of 6 to 11 but highly influenced by the nature of the metal ion (Ca^2+^, Fe^3+^, Hg^2+^ and Mn^2+^). For instance, the catalase-like activity of Co_3_O_4_ MNPs was inhibited by Fe^3+^ at pH 7.0 but enhanced at pH 10.0. Conversely, the nanozyme was enhanced by Mn^2+^ at pH 7.0 but inhibited at pH 10.0. This could provide a powerful approach to discriminate metal ions (Figure 13).

### 2.9. Summary

Nanozymes are highly promising materials for the determination of metal ions with high sensitivity and selectivity. The vast majority of the nanozymes were reliant on the peroxidase-like activity, which could be enhanced or inhibited in the presence of metal ions through amalgam formation or surface modifiers. However, those that are based on oxidase-like activity are more desirable since they do not rely on unstable H_2_O_2_ or its addition to environmental samples.

Nanozymes have demonstrated their applicability for in-field measurements where sample processing is conducted on-site. Most of the reported nanozymes are based on peroxidase-like activity and operate optimally under mildly acidic conditions (pH 4), requiring dilution in sodium acetate/acetic acid buffer solutions, with colour development ranging from a few minutes up to half an hour. Such sample processing can limit the practicality of in-field measurements, and although neutral pH operation is possible, this could be at the sacrifice of sensitivity. Nanozymes deposited on solid supports such as paper are attractive as they offer recyclability of the nanozyme, parallel sample measurements and signal amplification. Nowadays, advances in mobile phone technology have allowed the colour signal to be easily interpreted from built-in or custom-made applications, rather than relying on a UV–Vis spectrophotometer. However, accomplishing low detection limits with mobile phone technologies can be a challenge without extending the measurement time. Furthermore, approaches to more environmentally benign nanozymes or economically non-metal-based nanozymes are emerging. In particular, nanozymes that can serve multiple purposes, such as sensing and adsorption, are highly desirable in tackling environmental pollution.

## 3. Detection of Pesticides

### 3.1. Introduction

Pesticides have been extensively used in modern agriculture to control and eliminate pests by interfering with their metabolism, life cycle or behaviour. Their widespread use poses an enormous threat to human health and the ecosystem when released into the environment, food and water supplies. Exposure to pesticides is most likely through contact with crops or household products, via the skin, breathing or ingestion. Due to their high toxicity, environmental agencies have set maximum levels for pesticides in drinking and surface water [78].

Pesticides can be classified as insecticides, herbicides, fungicides or other types depending on their purpose, and they involve different classes of chemicals such as organophosphates, pyrethroids, carbamates, arsenic and nitrophenol derivatives [128]. Organophosphate pesticides (OPPs) are synthetic pesticides whose acute toxicity is associated with their ability to inhibit acetylcholinesterase (AChE) enzyme in the central nervous system, resulting in the accumulation of the neurotransmitter acetylcholine [129]. Symptoms of organophosphate exposure include headaches, nausea, diarrhoea and respiratory arrest.

Classical techniques for detecting pesticides include liquid or gas chromatography coupled with mass spectrometry (LC-MS, GC-MS) [130,131], which offer excellent sensitivity in the nanomolar range. However, they are not amenable to rapid on-field detection of pesticide residues and require operation by a highly trained technician. Enzyme activity inhibition methods are promising alternatives due to their ease in operation and rapid response. These assays rely on a change in enzyme activity upon exposure to pesticides. For instance, acetylcholinesterase and butyrylcholinesterase are irreversibly inhibited by OPPs, providing a means for indirect pesticide detection. A range of enzyme assays have been developed including colorimetric Ellman assays, electrochemical assays, fluorescence assays and chemiluminescence [132,133,134,135].

Enzymes also form the basis of bioremediation strategies to reduce the impact of pesticides in the environment by degrading and transforming pollutants into less toxic forms [136]. There are several types of enzymes involved in the detoxification of pesticides including oxidoreductases, hydrolases and lyases [136,137]. Oxidoreductase enzymes catalyse the transfer of electrons from one molecule to another and often require additional cofactors to act as electron donors, acceptors or both. Hydrolases are commonly involved in pesticide remediation by hydrolysing esters, peptide bonds, carbon–halide bonds, etc., and generally operate in the absence of redox cofactors, making them highly attractive for remediation. For example, alkaline phosphatase is a hydrolase enzyme responsible for removing a phosphate from organophosphate pesticides. Lyases are a smaller class of enzymes than oxidoreductases and lyases. They catalyse the cleavage of carbon–carbon bonds and carbon bonds with phosphorus, oxygen, nitrogen, halides and sulfate in the absence of redox cofactors and water. As bioremediation involves the use of microorganisms and their enzymes, several environmental parameters such as temperature, moisture content and pH can affect microorganism growth, which, in turn, can affect the rate of pollutant degradation.

### 3.2. Nanozymes for Pesticide Detection

Recent years have seen the emergence of nanozymes for the monitoring and degradation of pesticide residues on plants, crops, soil and water samples. Some of the strategies for pesticide detection are reliant on enzyme-like inhibition assays [138,139,140] or exploit the phosphatase-like activity for pesticide degradation [141,142,143]. Those that are based on the phosphatase-like activity are type I nanozymes involving nanoceria [141,142,143], whereas those based on the peroxidase-like activity are mainly type I Fe_3_O_4_ nanoparticles [140,144,145] or type II metal particles [138,146,147,148].

There are several studies which rely on inhibition of the oxidase or peroxidase-like activity of nanomaterials by pesticides. Nara and co-workers [138] have developed a sensitive and selective colorimetric assay for malathion detection using palladium–gold nanorods. The nanorods exhibit high peroxidase-like activity in the pH 2 to 6 range and better kinetic parameters than HRP. The peroxidase-like activity was quenched by the presence of malathion, with the OPD colour output being diminished. A low detection limit of 60 μg/L could be achieved, with no cross-reactivity from other analogous organophosphates or metal salts. Xia et al. [149] developed a colorimetric assay for pyrophosphate by exploiting the peroxidase-like activity of MoS_2_ quantum dots. By aggregating the quantum dots via the addition of Fe^3+^, the peroxidase-like activity could be enhanced. However, when pyrophosphate coexisted with Fe^3+^, the enhancement effect diminished due to the strong coordination between pyrophosphate and Fe^3+^. A detection limit of 1.82 μM pyrophosphate was able to be achieved. Kushwaha et al. [150] reported the oxidase-like activity of Ag_3_PO_4_ nanoparticles for the colorimetric detection of chlorpyrifos using TMB as a substrate. Ag_3_PO_4_ was able to oxidise chlorpyrifos to chlorpyrifos oxon and sulphide ions. The sulphide ions interacted with Ag_3_PO_4_ and inhibited the catalytic activity through a negative feedback loop. As a result, chlorpyrifos as low as 9.97 mg/L could be detected. In other work, Biswas et al. [146] have shown that the peroxidase-like activity of gold nanorods can be inhibited by malathion by interacting with the surface of the nanorods. They hypothesised that the positive charge of the nanorod surface coated with cetyltrimethylammonium bromide had a high affinity for the sulfanyl group of malathion. This interaction masked the enzymatic activity and enabled detection of malathion down to 1.78 mg/L. Other organophosphates such as chlorpyrifos and parathion lack a sulfanyl group; thus, the colorimetric assay for malathion was highly selective. Furthermore, potential interference from metal salts containing Zn^2+^, Pb^2+^, Co^2+^ or Mg^2+^ as sulfates or nitrates was less than 0.01%.

**Figure 14:**
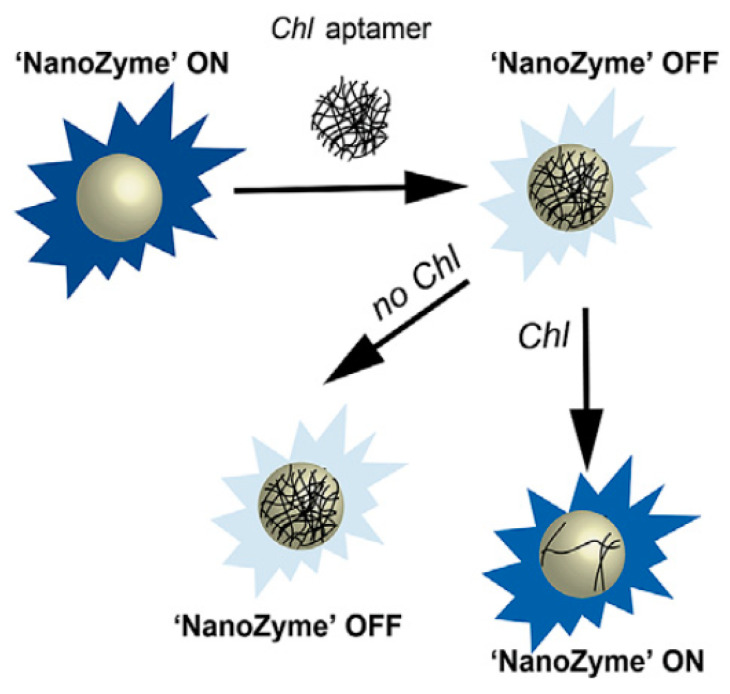
Working principle of tyrosine-capped silver nanoparticles used for the detection of chlorpyrifos (Chl). (Reprinted with permission from [147]. Copyright © 2019, Elsevier B.V. All rights reserved).

Highly specific strategies for pesticide detection using aptamers have been designed by Weerathunge et al. [147]. Firstly, they exploited the intrinsic peroxidase-like activity of tyrosine-capped silver nanoparticles. Subsequently, a chlorpyrifos-specific aptamer was incorporated onto the surface of the nanoparticles which switched the nanozyme to the “off” state. To realise sensing of chlorpyrifos, the nanozyme sensor was switched back to its “on” state due to aptamer desorption from the nanoparticle surface as a result of aptamer–chlorpyrifos binding (Figure 14). High specificity of the nanozyme sensor was afforded as the presence of other organophosphate pesticides did not lead to aptamer desorption and a detection limit as low as 11.3 mg/L was possible. Based on the same principle [148], tyrosine-capped gold nanoparticles as peroxidase-like nanozymes have also been used for the specific detection of acetamiprid. Compared to surface-enhanced Raman spectroscopy using silver dendrites [151], the detection of acetamiprid using nanozymes was five times more sensitive, with a detection limit of 0.1 mg/L.

**Figure 15:**
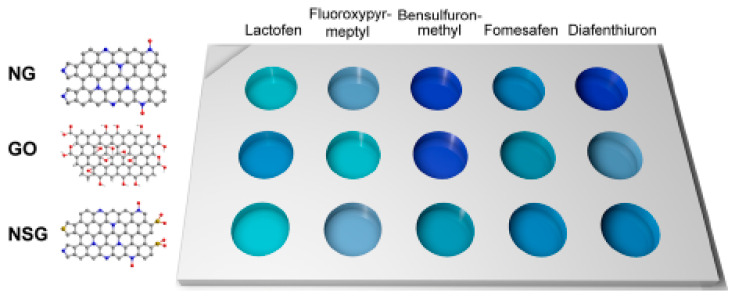
An array of peroxidase-mimicking graphene materials (graphene oxide (GO), nitrogen-doped graphene (NG) and sulphur-co-doped graphene (NSG)) used for the detection of five types of aromatic pesticides in the presence of TMB and H_2_O_2_. (Reprinted with permission from [139]. Copyright © 2020, American Chemical Society).

To target a suite of pesticides, nanozyme sensor arrays based on graphene oxide, nitrogen-doped graphene and sulphur-co-doped graphene with peroxidase-like activities have been developed (Figure 15) [139]. The interaction of the graphene materials with the aromatic pesticides lactofen, fluoroxypyr-meptyl, bensulfuron-methyl, fomesafen and diafenthiuron decreased their peroxidase-like activities. Molecular dynamics calculations confirmed that the enzyme-mimicking active sites (graphitic nitrogen in nitrogen-doped graphene, and carboxyl groups in graphene oxide) were blocked by the pesticides. Discrimination of the five pesticides (at 11 different concentrations) was demonstrated by using the three types of graphene prepared in a 96-well plate along with TMB and H_2_O_2_ in pH 4.0 sodium acetate buffer. By using linear discriminant analysis, the colorimetric response patterns were transformed into 2D canonical score plots which showed good clustering of the pesticides into five groups.

There have also been significant advances in using the peroxidase-like activity of Fe_3_O_4_ nanoparticles for the detection of pesticides. Guan et al. [144] have reported the chemiluminescent switching of Fe_3_O_4_ nanoparticles in the presence of pesticides. The nanoparticles catalyse the decomposition of dissolved oxygen to generate superoxide anions, enhancing the chemiluminescent intensity of luminol. The chemiluminescent signal could be quenched by the addition of ethanol as a radical scavenger; however, it was inhibited by a non-redox pesticide, ethoprophos. The strong surface coordinative reactions enabled the detection of ethoprophos down to 0.1 nM. Structurally analogous pesticides such as profenofos were also able to switch on the chemiluminescence, whereas the signal intensity from dylox and 2,4-dichlorophenoxyacetic acid was much lower. Therefore, the high specificity of the luminol-Fe_3_O_4_ system was attributed to the detection of organophosphorus esters with a P-S bond. Liang et al. [140] have also developed an assay for organophosphates based on Fe_3_O_4_ nanoparticles as a peroxidase mimic in combination with the enzymes AChE and choline oxidase (CHO). AChE and CHO catalyse the formation of H_2_O_2_ in the presence of acetylcholine, which can then be detected colorimetrically by Fe_3_O_4_ nanoparticles using TMB as a substrate. The reaction scheme is detailed in Equations (6)–(8). The presence of organophosphate compounds acephate and methyl-paraoxon as representative pesticides, and nerve agent Sarin, inhibited the activity of AChE and decreased the colour output. Compared to the traditional enzyme activity-based methods, the Fe_3_O_4_ peroxidase-like nanoparticles were more sensitive due to the catalytic activity of the nanoparticles, allowing concentrations as low as 1 nM Sarin, 10 nM methyl-paraoxon and 5 μM acephate to be detected. Boruah and Das [145] have successfully demonstrated that Fe_3_O_4_-TiO_2_/reduced graphene oxide nanocomposites could be used as a colorimetric assay for atrazine as well as photocatalytic degradation of atrazine. The nanocomposite was highly effective towards the oxidation of TMB at a pH of 3. However, the absorbance intensity at 652 nm was diminished by atrazine, which was attributed to hydrogen bonding between the pesticide and TMB. The detection limit for atrazine was found to be 2.98 μg/L. The degradation of atrazine was monitored by UV–Vis spectroscopy at 221 nm and it was shown that over 99% degradation was achieved within 40 min. This dual-responsive material is highly promising as it is also magnetically separable and could be recycled up to ten times.

(6) $${\mathrm{acetylcholine}+H}_{2}O\overset{\mathrm{AChE}}{\to }\mathrm{choline}$$

(7) $$\mathrm{choline}{+O}_{2}\overset{\mathrm{CHO}}{\to }H_{2}O_{2}$$

(8) $$H_{2}O_{2}+\mathrm{TMB}\overset{{\mathrm{Fe}}_{3}O_{4}}{\to }\mathrm{oxidised}\;\mathrm{TMB}$$

**Figure 16:**
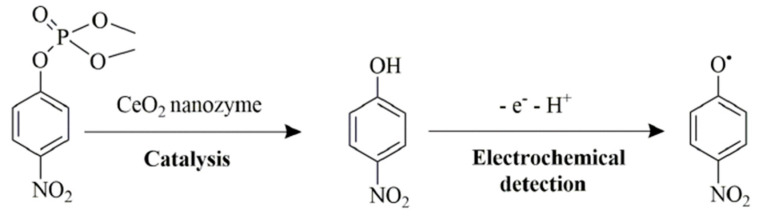
Schematic of nanoceria as a bifunctional material for catalysis and electrochemical detection of methyl-paraoxon. (Reprinted with permission from [143]. Copyright © The Authors. Distributed under a Creative Commons (CC BY-NC-ND 4.0) license).

Organophosphorus hydrolase is a useful phosphatase enzyme for detecting and degrading organophosphate pesticides with high specificity. As a degradation strategy, Wei et al. [141] have used nanoceria as a phosphatase mimic for the hydrolysis of organophosphate pesticides to *p*-nitrophenol using methyl-paraoxon as a representative compound. The hydrolysed product exhibited a bright yellow colour, which was analysed spectroscopically and with a smartphone. Under the optimal condition of pH 10, a detection limit of 0.42 μM was achieved. Dried plant samples were analysed by extracting the pesticide residues with ethyl acetate, evaporated to dryness and reconstituted in water. Potential interferents such as Na^+^, K^+^, Mg^2+^, glucose, alanine, ascorbic acid, sodium acetate and tyrosine were evaluated. All substances except ascorbic acid demonstrated negligible interference. The same researchers [142] have also combined the remarkable phosphatase-mimicking activity of nanoceria with carbon dots for the fluorometric determination of pesticides. Carbon dots are attractive as fluorescent probes as they exhibit low toxicity compared to conventional semiconducting quantum dots. The hydrolysis of methyl-paraoxon to *p*-nitrophenol is yellow and largely overlaps with the excitation spectra of carbon dots. Thus, fluorescence quenching of the carbon dots was observed by the generated *p*-nitrophenol. The limit of detection for *p*-nitrophenol was calculated to be 0.376 μM. Electrochemical detection of the degradation product of methyl-paraoxon has also been demonstrated using nanoceria. Sun et al. [143] have exploited the bifunctionality of nanoceria as a phosphatase mimic to degrade methyl-paraoxon to *p*-nitrophenol, followed by electrochemical detection of *p*-nitrophenol at a nanoceria-modified glassy carbon electrode (Figure 16). The electrochemical method was highly sensitive, with a methyl-paraoxon detection limit of 0.06 μM.

### 3.3. Summary

With further development, colorimetric assays can be configured for rapid, low-cost, in-field detection of pesticides. However, the sensitivity towards trace pesticide residues remains a significant challenge as most nanozymes can only achieve sub-micromolar detection limits. Strategies to amplify the signal and improve specificity include aptamer surface modification and the use of molecularly imprinted polymers. Electrochemical and fluorometric methods have also been explored as alternate sensitive transduction strategies.

Although the detection of pesticides is important, their removal is also paramount since they can last several years in the environment before breaking down. Nanozymes have emerged with the capability to breakdown or degrade pesticides into more benign products and will be covered in more detail in Section 5. This section has covered the use of nanozymes for the detection of pesticides, the most prevalent type of persistent organic pollutant. Section 4 will discuss methods to detect other types of persistent organic pollutants.

## 4. Detection of Other Persistent Organic Pollutants

**Figure 17:**
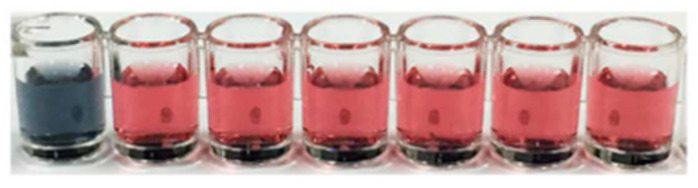
Selectivity of aptamer-modified gold nanoparticles for streptomycin. From left to right: streptomycin, amoxicillin, tetracycline, oxytetracycline, carbamazepine, diclofenac and penicillin added to aptamer-modified gold nanoparticles, ABTS and H_2_O_2_. (Reprinted with permission from [164]. Copyright © The Authors. Distributed under a Creative Commons (CC BY-NC) license).

Persistent organic pollutants are compounds that can persist in the environment for extensive periods and be transported by wind and water or through the food chain. They include organophosphorus compounds, phenolic compounds, dyes and antibiotics. As discussed in the previous section, organophosphorus pesticides are the most widely encountered persistent organic pollutant. Phenolic compounds such as chlorophenols and bisphenols are also broadly used as pesticides, including as wood preservatives and disinfectants [152], and are commonly detected in ground water and soil. They are a major cause for health concern as they can be carcinogenic, neurotoxic, affect the reproductive system and disrupt the endocrine system. For the detection of phenol [153], Barrios-Estrada used Pt_3_Au_1_ nanoparticles decorated with few-layer MoS_2_ nanosheets as peroxidase mimics. The oxidative coupling of phenol with 4-aminoantipyine in the presence of H_2_O_2_ resulted in the formation of a pink colour, with an optimum pH of 8.0–9.0. Nanozymes have also been shown to be highly promising candidates for the degradation of phenols [154,155,156,157,158] and will be discussed in Section 5. The efficiency in the removal and degradation of textile dyes will be covered in Section 5. Many organic dyes are toxic and not easily degraded in wastewater treatment plants [159]. These dyes serve as chromogenic substrates for the nanozymes, with the colour diminishing over time [160,161]. Antibiotics are classified as emerging pseudo-persistent organic pollutants as they are resistant to biodegradation due to their antimicrobial nature [162]. They are used in human and veterinary medicine and are mainly released into the environment through excretion [163]. Antibiotics are also used in agriculture, resulting in their presence in animal-derived food products, and can lead to serious side effects such as allergic reactions, hearing loss and kidney damage [164]. Zhao et al. [164] have developed an aptamer-modified gold nanoparticle sensor for the colorimetric detection of streptomycin. The gold nanoparticles exhibited peroxidase-like activity, which was diminished by coverage with streptomycin-specific aptamers. The presence of streptomycin as low as 86 nM resulted in re-establishment of the colorimetric signal from ABTS due to the formation of a streptomycin–aptamer complex. The sensor was highly specific, with little interference from tetracycline, oxytetracycline, carbamazepine, penicillin, amgoxicillin and diclofenac. Despite the gold nanoparticles appearing red in colour, a greyish-green colour was well observed from the streptomycin sample (Figure 17). Sharma, Bansal and co-workers [165] have similarly exploited the high specificity of ssDNA aptamers coupled with the intrinsic peroxidase-like activity of gold nanoparticles for the detection of kanamycin. A rapid visual readout was possible within 3–8 min, with a detection limit of 1.49 nM.

## 5. Environmental Remediation with Nanozymes

### 5.1. Introduction

Human activities have resulted in significant contamination of our environment [3,166,167]. Many toxic pollutants, including heavy metals, dyes, other organic and inorganic substances, are generated during the production of items for our consumption and also during their use. These include pesticides, pharmaceuticals and clothes. Due to a lack of regulation and awareness, many toxic compounds have been discharged into the environment, particularly aquatic environments (lakes, rivers and oceans) without a second thought. These pollutants have caused significant damages to our ecological systems, including humans, plants, animals and microbes. They have been established to be the cause of toxic and carcinogenic effects on humans through contamination of drinking water and foods [167]. Significant efforts have been devoted to the remediation work to remove these pollutants and/or degrade them to less harmful products [92,166,168].

Methods for the removal of contaminants from aquatic environments include adsorption, membrane filtration, distillation, oxidation, biocatalytic and photocatalytic degradation [166,168]. Biocatalytic methods (enzymatic and microbial) have been investigated as effective means for the degradation of organic pollutants. The advantages of these biocatalytic methods are that they can be operated under mild and natural environments. Additionally, microbes and enzymes themselves could decompose into benign compounds when their mission is completed, thus eliminating unwanted environmental pollution from the treatment agent itself [136,137]. However, microbes and enzymes can only be functional and effective in narrow thermal and pH windows. The cost of producing enzymes, their lack of recyclability and the fact that biodegradation can be quite slow have hindered their large-scale, widespread application in environmental pollutant remediation [92,166].

Efforts to overcome the limitations of enzymatic methods have driven investigations into using nanozymes as remediating agents for environmental pollutants. Nanozymes have been shown to demonstrate catalytic properties, e.g., peroxidase- and oxidase-like, which are utilised in the degradation of pollutants by natural enzymes. Nanozymes could overcome enzyme limitations in terms of cost of production, recyclability, higher rate of reactions and wider operational windows (pH and temperature) [3,92,168]. The previous section has shown that nanozymes are effective for the detection of heavy metals and organic pollutants in the environment. Nanozymes could also be used to degrade environmental pollutants. In this section, applications and future prospects of nanozymes in environmental pollutant remediation will be discussed, focusing on the degradation of persistent organic pollutants.

Persistent organic pollutants include phenolic compounds, pesticides, dyes and organophosphorus compounds [169]. Phenolic compounds, particularly chlorophenols, have been widely used in pesticides, dyes and other synthetic compounds [156,168,170]. Chlorophenol pollutants can cause serious problems for the environment as they are highly toxic and resistant to chemical and biological degradation in the environment. These compounds are classified as top priority pollutants by the US EPA (Environmental Protection Agency) and other environmental regulators around the world. Current methods for phenol removal include solvent extraction, physical adsorption, pervaporation, wet air oxidation, ozonolysis, wet peroxide oxidation, electrochemical oxidation, photocatalytic oxidation, supercritical water gasification, electrical discharge degradation and bio-degradation [156]. Nanozyme-mediated degradation of phenolic compounds is a highly promising method with significant advantages over current methods [168].

There are many types of textile dyes currently in use (including phenolic dyes), and many of these are recalcitrant and toxic compounds that are not easily degraded in wastewater treatment plants [159]. On a global scale, approximately 700,000 tonnes (in 2005) of textile dyes are consumed yearly [171]. Textile manufacturing activities are currently concentrated in developing countries, where the treatment of organic dyes will need to be low-cost to be more widely applicable. Here, nanozymes could have significant advantages due to the low cost and high recyclability of certain nanozyme materials such as magnetic Fe_3_O_4_.

Organophosphorus compounds (OPPs, chemical warfare nerve agents and flame retardants), particularly OPPs, widely exist in the environment [136,172,173]. The increased use of highly toxic OPPs has made these compounds major contaminants in water, fruit and vegetables. OPPs have highly detrimental effects on human health and hence a method to remove them from the environment is invaluable [141].

Herein, the application of nanozymes in organic pollutant remediation will be discussed according to the nanozyme type classifications as proposed in the Introduction of this review.

### 5.2. Type I Nanozymes: Active Metal Centre (of Metalloenzyme) Mimics

Table 3 lists recent studies of type I nanozymes for the remediation of environmental pollutants. Since the report by Gao and co-workers [6] that Fe_3_O_4_ MNPs possessed enzymatic-like activity similar to naturally occurring peroxidases, they have been widely used for the oxidation of organic substrates as a detection method in the treatment of wastewater. Iron oxide nanoparticles, especially Fe_3_O_4_ MNPs, have been most widely investigated for the degradation of various environmental pollutants due to their peroxidase-like activity and the fact that Fe_3_O_4_ MNPs could be conveniently prepared from cheap and abundant precursors [168].

As can be seen in Table 3, the overwhelming enzyme-like catalytic activity marshalled by type I nanozymes in the degradation of phenol compounds and dyes is the peroxidase-like activity. Of the 17 examples listed above, only two examples where MnO_2_ nanoparticles and Cu complex displayed laccase-like activity are non-peroxidase-like examples [172,173]. Laccases promote the oxidation of phenolic compounds with the reduction of oxygen to water. Fe_3_O_4_ was the most common material of the metal oxides investigated for environmental pollutant degradation.

**Figure 18:**
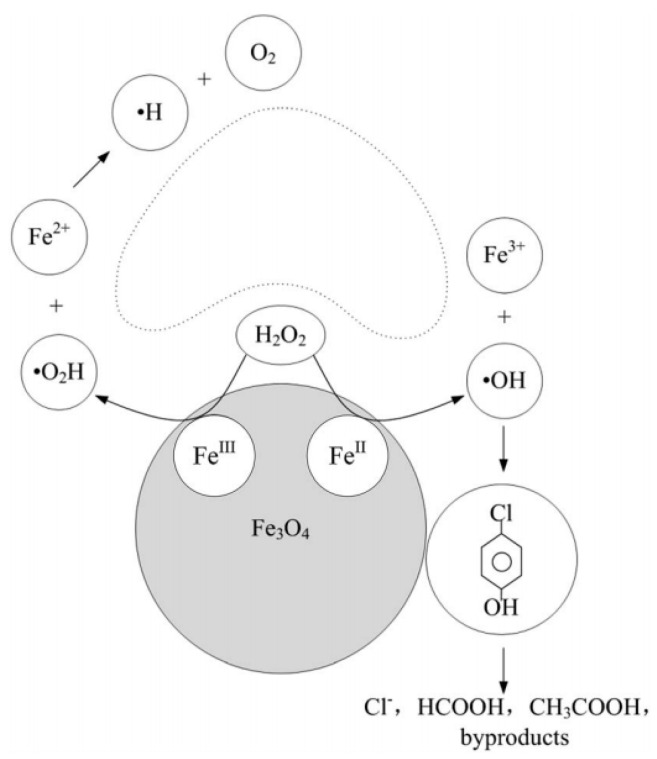
Illustration of the mechanism of catalytic oxidation of 4-chlorophenol by Fe_3_O_4_ nanoparticles. (Reprinted with permission from [75]. Copyright © 2019 WILEY-VCH Verlag GmbH & Co. KGaA, Weinheim, Germany).

The first example of using ferromagnetic nanoparticles to facilitate the decomposition of phenols was reported in 2008 [154]. Phenols were removed from wastewater by the peroxidase activity of the Fe_3_O_4_ nanoparticles. The hydroxyl radical formed in the reaction between Fe_3_O_4_ and hydrogen peroxide catalytically degraded phenols. More than 80% of phenols were removed at 16 °C and pH 3 and the nanozyme could be reused several times. Fe_3_O_4_ MNPs (5.7 nm in size) could also effectively degrade and mineralise 2,4-dichlorophenol. Fe_3_O_4_ MNPs were effective as heterogeneous sono-Fenton catalysts for the degradation of bisphenol A (BPA) in a reasonably wide pH range of 3–9. However, the rate of the degradation was still too slow for practical application [174]. Magnetic Fe_3_O_4_ nanoparticles at 30 nm in size were demonstrated to be highly effective for the degradation of 4-chlorophenol by Cheng et al. [75]. In the presence of hydrogen peroxide and Fe_3_O_4_, 4-chlorophenol was degraded to Cl^−^, HCOOH (formic acid), CH_3_COOH (acetic acid) and by-products. Adsorption of 4-chlorophenol onto the iron oxide particle surfaces was shown to be only minimal (around 10%) and an acidic pH of 5 was the optimal pH for the highest activity. These Fe_3_O_4_ nanoparticles could be reused with no decrease in reactivity. In fact, an increase in their reactivity for the degradation of 4-chlorophenol was observed. A plausible mechanism was proposed for the catalytic degradation of 4-chlorophenol (Figure 18). Fe_3_O_4_ converted H_2_O_2_ via its peroxidase-like activity, to form the highly active hydroxyl radical HO^•^, which, when reacted with 4-chlorophenol, triggered a chain of reactions leading to the formation of chloride ion, formic acid, acetic acid and other by-products.

**Table 3:** Examples of type I nanozymes in environmental pollutant remediation.

Nanozyme	Pollutant	Enzyme-Like Activity	Year	Ref.
Fe_3_O_4_ MNPs	Phenol	Peroxidase	2008	[154]
Fe_3_O_4_ MNPs	Rh B	Peroxidase	2010	[175]
Fe_3_O_4_ MNPs	Sulfamonomethoxine	Peroxidase	2011	[176]
Humic acid-Fe_3_O_4_ MNPs	Sulfathiazole	Peroxidase	2011	[177]
Fe_3_O_4_ MNPs	BPA	Peroxidase	2012	[174]
Fe_3_O_4_ MNPs	2,4-Dichlorophenol	Peroxidase	2012	[178]
CuO porous structures	Phenol	Peroxidase	2014	[155]
Fe_3_O_4_ MNPs	4-Chlorophenol	Peroxidase	2015	[170]
Fe_3_O_4_ nanorod bundles	Crystal violet	Peroxidase	2015	[160]
CuO nanoparticles	Phenol	Peroxidase	2015	[156]
Fe_2_O_3_·0.5H_2_O (ferrihydrite) and hematite (Fe_2_O_3_)	Methylene blue	Peroxidase	2016	[161]
VO_x_ nanoflakes	Rh B	Peroxidase	2016	[179]
MnO_2_ nanomaterials	ABTS	Laccase	2017	[172]
MNPS@chitosan	Phenol	Peroxidase	2018	[157]
Fe_3_O_4_ nanorods	Rh B, methylene blue and methyl orange	Peroxidase	2019	[180]
Cysteine-histidine Cu	2,4-Dichlorophenol and other phenolic compounds	Laccase	2019	[173]
CeO_2_ nanoparticles	Rh B, fluorescein, xylene cyanol FF, Brilliant Blue G-250, and Coomassie Brilliant Blue R-250	Peroxidase	2020	[181]

Fe_3_O_4_ MNPs tend to aggregate and hence lead to the reduction of catalytic activity. To increase the catalytic ability and stability of Fe_3_O_4_ MNPs, Jiang and co-workers [157] synthesised ferromagnetic chitosan (MNP@chitosan, an alkali polysaccharide) nanozyme with particle sizes of around 12 nm. The MNP@chitosan was shown to be effective for the decomposition of phenol. The optimum pH for this catalytic activity was pH 4. The MNP@chitosan was also found to be recyclable; however, the degradation rate slowly decreased with each reuse cycle.

Fe_3_O_4_ MNPs or nanorods have also been successfully used in the degradation of Rhodamine B (Rh B) [175], crystal violet [160,180], methylene blue [161,180] and methyl orange [180] in the presence of hydrogen peroxide with peroxidase activity. Humic-acid-coated ferromagnetic nanoparticles (Fe_3_O_4_ 10–12 nm in size) were effective for the removal of sulfathiazole from aqueous media in the presence of H_2_O_2_. The catalytic efficiency was higher at lower pH (as low as pH 3.5) and higher temperatures, up to 60 °C [177].

Fe_3_O_4_ MNPs can activate persulfate anion S_2_O_8_^2−^ to generate the powerful sulfate radical SO_4_^●^**^−^**, which has high potential for the degradation of organic pollutants. The method was demonstrated to degrade sulfamonomethoxine (an antibiotic) in the presence of S_2_O_8_^2−^ [176].

Ferric oxides (Fe_2_O_3_) such as 2-line ferrihydrite (2LFh, Fe_2_O_3_·0.5H_2_O, average size 5 nm) and hematite (Fe_2_O_3_, average size 100 nm) could also catalyse the degradation of methylene blue [161]. The more highly crystalline hematite was found to be more effective in degrading methylene blue than ferrihydrite. The author concluded that the ordered crystal planes of the hematite nanoparticles had a greater influence on the catalytic activity than the high surface area of 2LFh nanoparticles. A slightly basic pH of 8 was the optimal pH.

Other metal oxide nanoparticles have also shown peroxidase-like activity in the degradation of organic pollutants. Examples include CuO [155,156], CeO_2_ [181], MnO_2_ [172] and VO_x_ [179].

CuO micro-/nanostructures with clean surfaces were prepared and demonstrated to have high peroxidase activity using TMB as the model substrate. These CuO structures could also degrade phenol, illustrating their promise as a reagent for wastewater treatment [155]. Feng and co-workers have shown cupric oxide nanoparticles to be highly efficient in the degradation of phenol, catechol, hydroquinone and other by-products [156]. Larger particles (30 nm) were found to be inefficient for phenol degradation. While no explanation was provided, the enhanced surface area to volume in smaller particles is likely to be a contributing factor. The optimal pH range was 3–7; above pH 7, the catalytic activity dropped off quite rapidly. In a highly acidic environment (pH 2), the phenol removal efficiency was very low as CuO particles dissolved to Cu^2+^ ions, which have low peroxidase activity.

**Figure 19:**
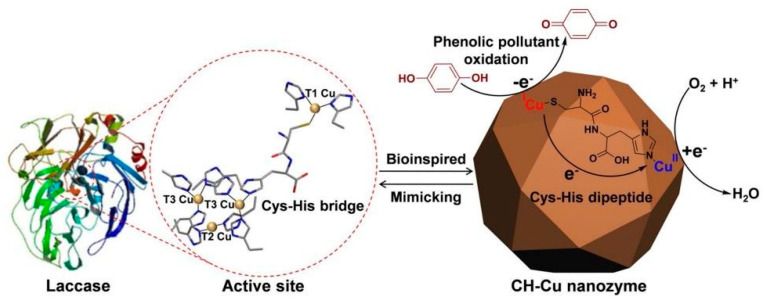
Cysteine-histidine Cu nanozyme for the oxidation of phenolic pollutant. (Reprinted with permission from [173]. Copyright © 2019, Elsevier B.V. All rights reserved).

Wang et al. [173] constructed a laccase-mimicking nanozyme by coordinating Cu^+^/Cu^2+^ to cysteine-histidine dipeptide to form an inorganic polymer CH-Cu which possessed good catalytic efficiency for the degradation of chlorophenol and bisphenol in the absence of H_2_O_2_ (Figure 19). CH-Cu was also highly robust and could operate at pH 3–9, temperatures −20 to 90 °C, high salinity and could be stable for more than 3 weeks. The CH-Cu nanozyme could be reused multiple times.

Wang and co-workers showed that the degradation of various organic dyes such as Rh B, fluorescein and Brilliant Blue G-250 in the dark by CeO_2_ could be enhanced using fluoride ions at low-pH conditions [107].

**Figure 20:**
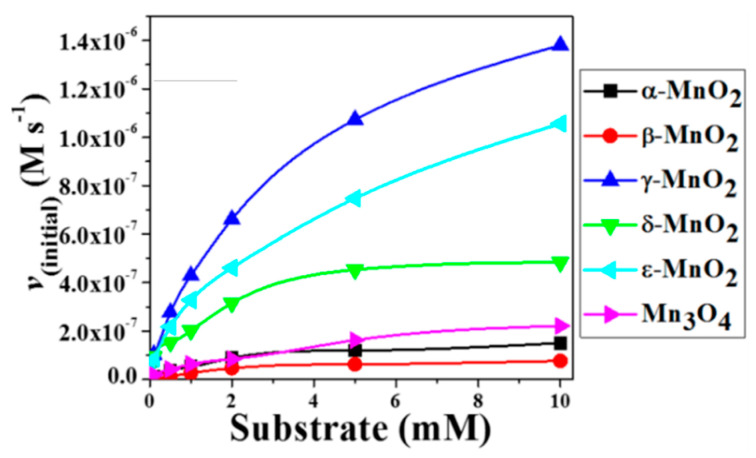
Degradation of ABTS with different manganese oxide materials. (Reprinted with permission from [172]. Copyright © The Authors. Distributed under a Creative Commons (CC BY) license).

Manganese oxide, MnO_2_, nanomaterials were demonstrated to have laccase-like catalytic reactivity and could catalyse the oxidation of pollutants in wastewater treatment in the absence of H_2_O_2_. ɣ-MnO_2_ was the most efficient catalyst for the degradation of ABTS and 17-β-estradiol (E2) amongst the different manganese oxide nanomaterials tested [172] (Figure 20).

**Figure 21:**
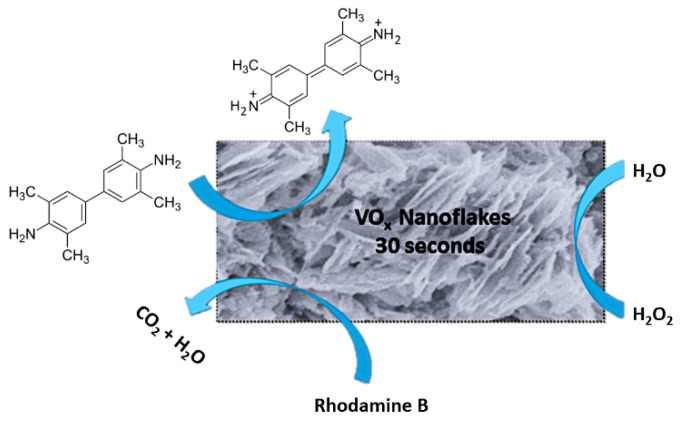
VO_x_ nanoflakes as efficient catalysts for the oxidation of TMB and decomposition of Rh B. (Adapted with permission from [179]. Copyright © 2016, American Chemical Society).

Zeb et al. [179] reported that mixed-phase VO_x_ nanoflakes could be conveniently prepared in a single step and be a highly effective Fenton reagent, which could fully decompose Rh B within 60 s (Figure 21). The VO_x_ nanoflake also possessed peroxidase-like activity and could oxidise TMB efficiently, with *V*_max_ of approximately 27 times more than HRP. These results indicated that VO_x_ nanoflakes could be used for the effective degradation of environmental pollutants.

### 5.3. Type II Nanozymes: Functional Mimics

There are few examples of type II nanozymes that have been shown to degrade environmental organic pollutants (Table 4). Peroxidase-like activity was the dominant activity observed [182,183,184]; however, oxidase-like activity [185] was also observed.

Xu et al. [183] investigated Fe(0) particles as heterogeneous Fenton-like catalysts for the removal of 4-chloro-3-methyl phenol. It was found that lower pH led to higher catalytic activity and the reaction was still quite efficient up to pH 6.1. A range of intermediates and products were observed by LC-MS, and the final products after 60 min of reaction included chloride ion, oxalic acid, acetic acid and formic acid. The catalytic activity decreased with time and the recyclability of the material was not demonstrated. It is worth noting that Fe(0) was oxidised to Fe(IV) (FeO^2+^) during the catalytic reaction; hence, the Fe(0) particles are best considered to be a pre-catalyst in this work.

In a communication, Safavi and co-workers [182] reported the preparation of carbon nanodots using a microwave-assisted method in ionic liquids. These carbon nanodots could degrade azo dyes methyl red and methyl blue via their peroxidase-like activity.

Chen et al. [184] showed that cubic boron nitride possessed peroxidase-like activity and could oxidise TMB. The cubic boron nitride could be reused multiple times. The catalytic activity was highest at acidic pH 5 and at temperatures around 40–50 °C. Cubic boron nitride also catalysed the degradation of Rh B in the presence of H_2_O_2_.

Zhang and co-workers [185] reported modified graphitic carbon nitride as a metal-free nanozyme which has dual oxidase–peroxidase functions as a cascade photocatalyst for the oxidation of TMB. Whilst the work was not directed towards applications in environmental pollutant degradation, the fact that TMB (a substrate in the colorimetric test that has a similar structure to many organic dyes) is efficiently oxidised to its blue form is a strong indication that the graphitic carbon nitride is likely to be effective in dye removal. The bifunctional oxidase–peroxidase activities of the material would be highly advantageous in large-scale applications as there would be no need for hydrogen peroxide.

Noble metal nanozymes have been shown to be applicable for the detection of environmental pollutants and in medicinal diagnosis. They have not been widely applied in the degradation of environmental pollutants. The high cost of noble metals is likely to be a prohibitive factor in the application of noble metal nanozymes for environmental pollutant remediation. Consequently, there have not been many studies on using noble metal (Au, Pt etc.)-based nanozymes for environmental pollutant remediation.

### 5.4. Type III Nanozymes: Nanocomposites

Recently, nanocomposite materials including metal/metal oxides on carbon materials, MOFs and bimetallic alloys (core-shell) have been investigated extensively as agents for environmental remediation. As shown in Table 5, peroxidase-like activity is the predominant catalytic activity utilised by composite nanozymes in the degradation of organic pollutants. The recalcitrant organic pollutants investigated in these studies included phenolic compounds [48,158,186,187,188,189], dyes [159,190,191,192,193,194,195,196] and organophosphorus compounds [197,198].

Magnetic Fe_3_O_4_ on carbon materials [48,186,187,188,191,192,195,199,200,201,202] are the most studied class of composite nanozymes for environmental degradation. Carbon materials by themselves have been used in wastewater treatment for a long time. Composites of Fe_3_O_4_ with carbon materials are expected to deliver significant benefits such as recyclability and dual adsorptive–catalytic activities in pollutant degradation applications. The excellent review by Ribeiro et al. [202] is an important source of information for readers who have special interest in the application of hybrid magnetic carbon nanocomposites for the degradation of organic pollutants in water treatment.

Different kinds of carbon materials have been investigated as nanocomposites with metal/metal oxide nanoparticles. Examples include carbon nanotubes, multiwalled carbon nanotubes (MWNTs), graphene, graphene quantum dots, graphene oxide, mesoporous carbon, etc. [202]. Zuo et al. [186] synthesised Fe_3_O_4_ MNP/MWNT nanocomplexes with peroxidase-like activity. In the presence of H_2_O_2_, the nanocomplex could efficiently catalyse the oxidative degradation of phenols to insoluble polyaromatic products that could be easily separated from aqueous solutions. Magnetically recoverable Fe_x_O_y_-MWNT was active in the degradation of Orange G dye [199]. The Fe_3_O_4_ nanoparticles (7 nm in size) that were grown on carbon nanotubes were shown to have higher catalytic reactivity as an enzyme mimic for the decomposition of the dye Orange II than Fe_3_O_4_ nanoparticles [195].

Peng [187] reported the synthesis of graphene-templated formation of ultrathin (2.1 nm) 2D lepidocrocite *ɣ*-FeOOH and showed that these nanostructures could catalyse the degradation of phenol in the presence of H_2_O_2_. Nanocomposites between graphene oxide quantum dots (graphene sheets with lateral sizes less than 100 nm) and Fe_3_O_4_ nanoparticles were also shown to be effective for the degradative removal of phenolic compounds via peroxidase-like reactivity [48]. The high reactivity of the nanocomposite (higher than HRP) was attributed to the unique properties of the graphene oxide quantum dots, namely high electron conjugation and better aqueous dispersion ability compared to graphene oxide sheets, and the synergistic interactions between graphene oxide quantum dots and Fe_3_O_4_ MNPs. Fe_3_O_4_/reduced graphene oxide [200] and Fe_3_O_3_/graphene oxide [201] were reported to be efficient for the degradation of methylene blue and Rhodamine B at neutral pH, respectively. In a report by Zubir and co-workers [192], graphene oxide–Fe_3_O_4_ nanocomposites were found to be efficient in the degradation of acid orange dye. In comparison with Fe_3_O_4_ MNPs, the Fe_3_O_3_/graphene oxide had similar reactivity in the first 45 min of the catalytic cycle. However, while Fe_3_O_4_ was completely deactivated after one use, the nanocomposites retained good reactivity for the entire course of the reaction.

**Figure 22:**
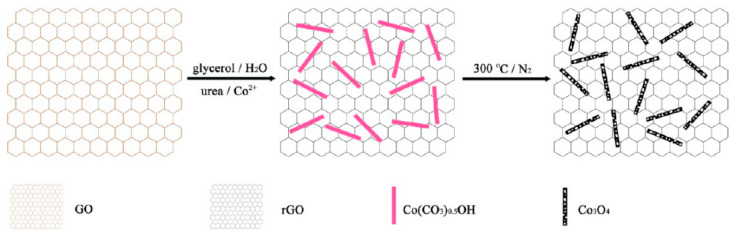
Procedure used in the preparation of porous Co_3_O_4_ nanorod-reduced graphene oxide, PCNG. (Reprinted with permission from [191]. Copyright © 2013, American Chemical Society).

Porous Co_3_O_4_ nanorod-reduced graphene oxide (PCNG) (Figure 22) was shown to have high peroxidase activity in the degradation of methylene blue [191]. The improved catalytic activity of the nanocomposites could be the result of the synergy between the functions of porous Co_3_O_4_ nanorods and reduced graphene oxides. Here, due to the π-π stacking between methylene blue and the aromatic areas of reduced graphene oxide, methylene blue was more easily adsorbed on the surface of the PCNG. Electrons from methylene blue are donated to the PCNG, which leads to an increase in electron density and mobility in the PCNG. Electron transfer from PCNG to H_2_O_2_ is accelerated, thus increasing the reaction rate of methylene blue oxidation by H_2_O_2_. The PCNG had high thermal stability and was stable in the presence of an organic solvent, e.g., ethanol, tetrahydrofuran and *N,N*-dimethylformamide.

**Figure 23:**
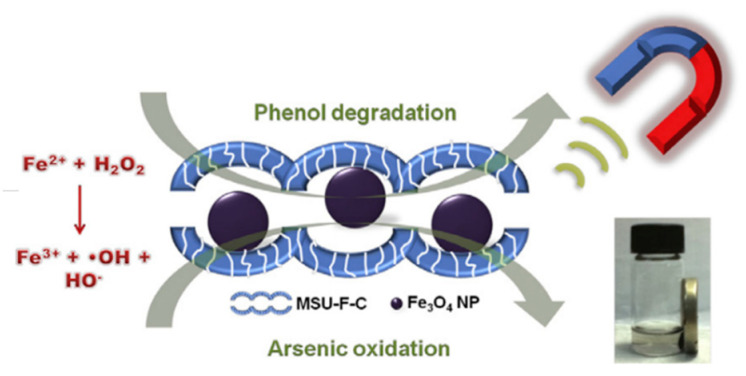
Removal of arsenic and phenol using Fe_3_O_4_/MSU-F-C as a catalytic adsorbent. (Reprinted with permission from [188]. Copyright © 2012 Elsevier Ltd. All rights reserved).

Chun et al. [188] showed that magnetite (Fe_3_O_4_)-loaded meso-cellular carbonaceous material, Fe_3_O_4_/MSU-F-C, was very efficient in the Fenton-like reaction as well as an adsorbent for the removal of phenol and arsenic (Figure 23). The material could be easily separable by applying a magnetic field due to its strong magnetic property.

In short, the increase in the performance of nanocomposites between magnetically separable iron species (and other metal species) and carbon-based materials was attributed to several synergistic effects which include: (i) the pollutant molecules are brought closer to the active sites by the increased adsorptive interactions of the carbon phase; at the active sites, strongly oxidising HO^•^ radicals are generated and react with the pollutant. The HO^•^ radical would have lower probability of partaking in the non-productive parasitic reaction with H_2_O_2_, thus increasing the efficiency of H_2_O_2_ consumption; (ii) iron–carbon nanocomposites usually have good structural stability and lower leaching of metal species due to the confinement effect imposed by the carbon phase; (iii) the regeneration of the active sites is enhanced either by electron transfer features or delocalisation of π electrons of the carbon-based materials; (iv) the active sites are highly dispersed as the result of the high specific area of the carbon phase; and (v) some carbon materials also have peroxidase-like activity on their own [202]. Several of these nanocomposites have been shown to be effective for environmental pollutant degradation at neutral pH [199,200,201,202], which is a significant advantage as the treated water (in a wastewater treatment plant) would not be required to undergo a neutralisation step.

**Figure 24:**
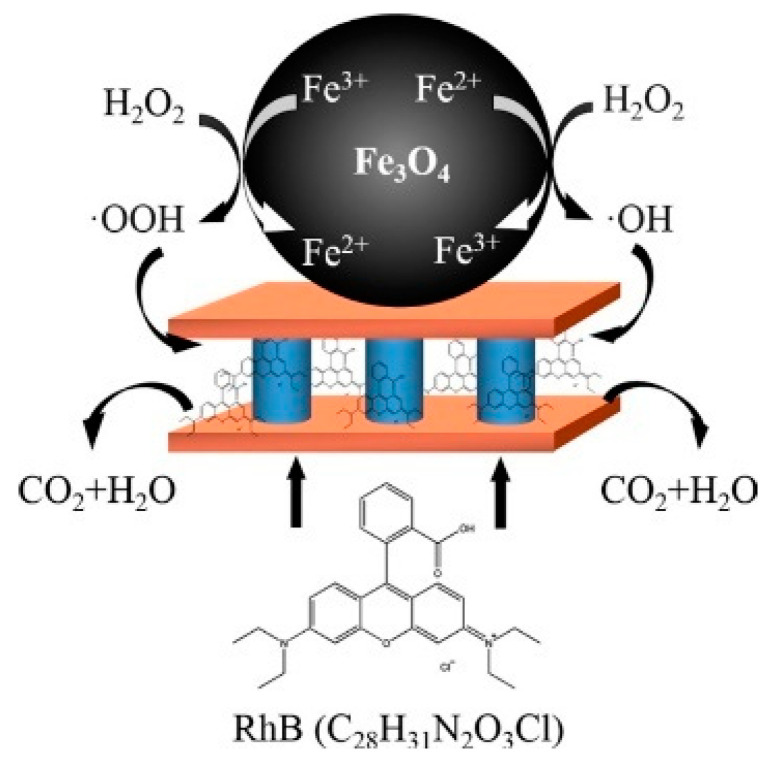
Fe_3_O_4_-decorated Al-pillared bentonite (Fe_3_O_4_/Al-B) as a peroxidase-like nanozyme for the degradation of Rh B. (Reprinted with permission from [194]. Copyright © 2015 Elsevier B.V. All rights reserved).

In addition to carbon materials, other materials have also been utilised in the preparation of nanocomposites for environmental pollutant degradation. Wan et al. [194] showed that Fe_3_O_4_ nanoparticle-decorated Al-pillared bentonite (Fe_3_O_4_/Al-B) had higher ability for the adsorption and degradation of Rh B than bare Fe_3_O_4_ (Figure 24). The composite also showed high stability and could be conveniently recycled.

**Figure 25:**
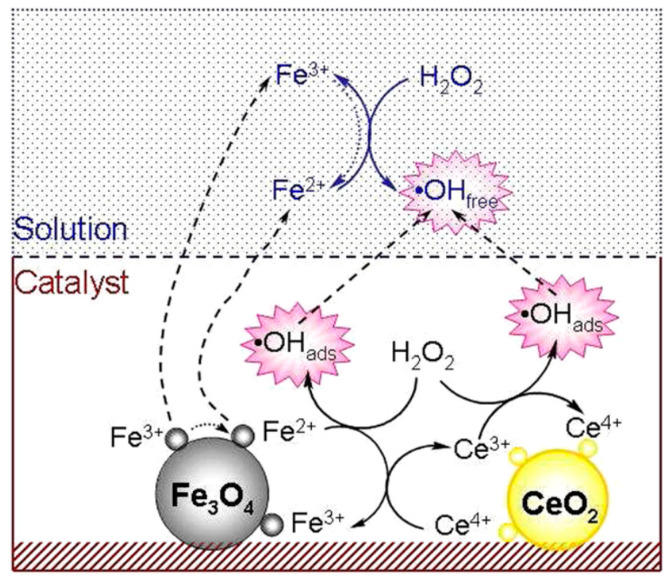
Proposed reaction mechanism of the H_2_O_2_ activation by Fe_3_O_4_/CeO_2_ catalyst under acidic pH. (Reprinted with permission from [158]. Copyright © 2012, American Chemical Society).

Fe_3_O_4_/CeO_2_ nanocomposites (5–10 nm in size) were reported by Xu and co-workers [158] to be effective for the degradation of 4-chlorophenol at acidic pH of 3 in the presence of H_2_O_2_ via the peroxidase-like activity. 4-Chlorophenol was decomposed by the HO^•^ radical including surface-bound and in-solution radicals. The material could be reused several times. The proposed mechanism for the generation of hydroxyl radical HO^•^ from H_2_O_2_ and the interactions between different oxidation states of iron and cerium ions to facilitate HO^•^ formation is given in Figure 25. Janoš et al. [198] synthesised magnetically separable composites consisting of Fe_2_O_3_ grains and CeO_2_ nanocrystalline surface (CeO_2_/c-Fe_2_O_3_) and applied them as a “reactive sorbent” for the decomposition of dangerous organophosphorus compounds including organophosphorus pesticide parathion-methyl.

**Figure 26:**
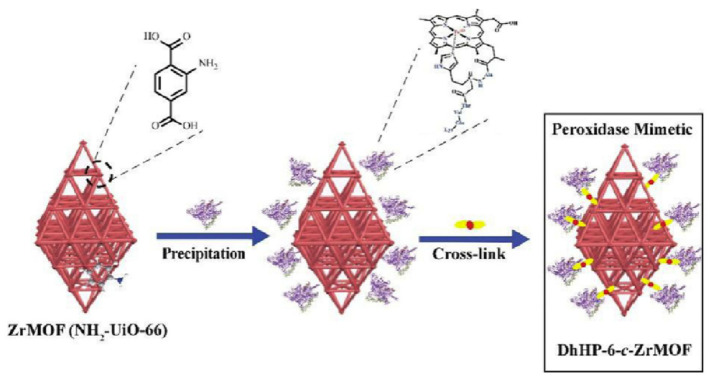
Schematic illustration of the synthesis of DhHP-*c*-ZrMOF. (Reprinted with permission from [189]. Copyright © 2019 Elsevier B.V. All rights reserved).

Deuterohemin-peptide conjugated onto metal–organic framework, DhHP-*c*-ZrMOF, acted as a peroxidase mimetic catalyst for the degradation of phenol [189]. The schematic summary of the synthetic process is outlined in Figure 26. It was found that the hemin attached onto the MOF was significantly more active for the degradation of phenol and could operate in a wider pH range.

**Figure 27:**
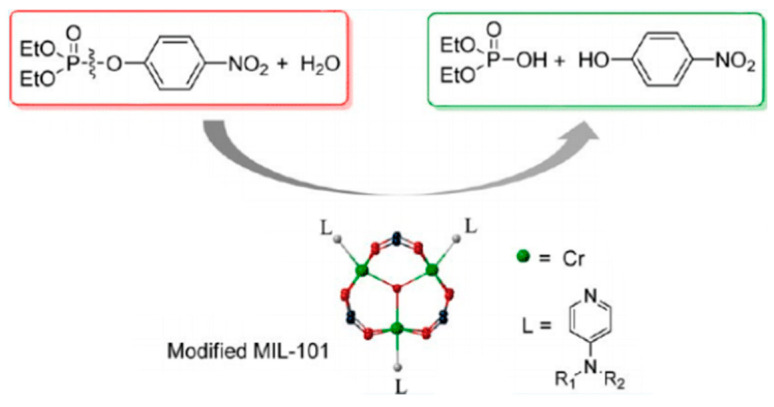
MIL-101 with chelated aminopyridines as a catalyst for organophosphorus ester degradation. (Reprinted with permission from [197]. Copyright © 2013, American Chemical Society).

Chromium(III) terephthalate metal organic framework (MIL-101) was demonstrated to be effective in facilitating organophosphorus ester degradation using paraoxon as the model substrate [197]. The MIL-101 had an optimal activity at a basic pH of 10. The degradation of paraoxon (Figure 27) generates two acidic products which are likely to be removed in basic pH by the reaction with hydroxyl functional group.

Li and co-workers [196] showed that the active sites in MOF-derived homobimetallic hollow nanocages are highly efficient as multifunctional nanozyme catalysts for biosensing and organic pollutant degradation. They fabricated Co-based homobimetallic hollow nanocages (HNCs)(C−CoM−HNC, M = Ni, Mn, Cu and Zn) and showed that the material acted as an efficient nanozyme for biosensing based on the excellent oxidase-like activity and for the degradation of an organic pollutant (Rh B).

Examples of other types of composite nanozymes were also reported. Polypyrrole/hemin nanocomposites were prepared and shown to have biosensing, dye removal ability and photothermal therapy [193]. The dye removal ability is not related to the catalytic activity of the nanocomposite and was established by the author to be an adsorbent effect. Cellulose-incorporated iron oxide magnetic nano-biocomposites as a peroxidase mimic have the potential to be a low-cost, recyclable option for the remediation of textile dyes, using the azo dye methyl orange as well as a textile effluent [159]. Niu et al. [203] prepared alginate/Fe@Fe_3_O_4_ core/shell structured nanoparticles (Fe_3_O_4_@ALG/Fe MNPs) for the defluorination and removal of norfloxacin (a fluoroquinolone antibiotic). The Fe_3_O_4_@ALG/Fe MNPs have higher efficiency for norfloxacin degradation compared with the Fe_3_O_4_ nanoparticle–H_2_O_2_ system, with 100% of the norfloxacin removed within 60 min. Mixed metal oxide, iron molybdate (Fe_2_(MoO_4_)_3_), was prepared and shown to act as a heterogeneous Fenton-like catalyst for the degradation of the dye Acid Orange II. The heterogeneous catalyst worked efficiently in a relatively wide pH range of 3 to 9. Good mineralisation of Acid Orange II was achieved [190].

### 5.5. Type IV Nanozymes: 3D Structural Mimics

There are only a limited number of studies using type IV nanozymes for the degradation of environmental pollutants (Table 6). The technical challenge in the construction of these 3D nanostructures is likely to be a contributing factor to this limitation.

**Figure 28:**
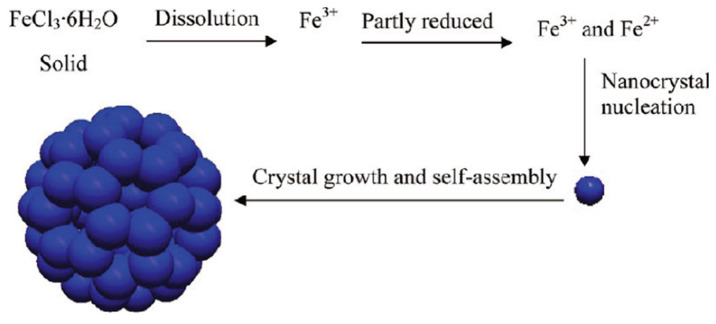
Preparation of porous Fe_3_O_4_ nanospheres. (Reprinted with permission from [204]. Copyright © 2011, American Chemical Society).

Zhu and Dao [204] prepared porous Fe_3_O_4_ nanospheres (Figure 28) and showed that they were highly effective as catalysts for the degradation of xylenol orange with H_2_O_2_ as the oxidant in aqueous solution. The porous Fe_3_O_4_ could be recycled multiple (7) times, with only a slight drop in activity after each cycle.

**Figure 29:**
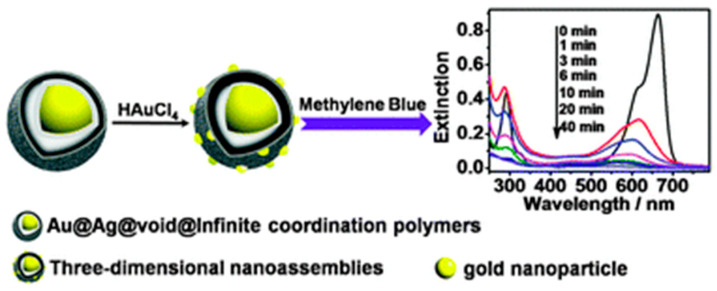
Three-dimensional assembly of gold nanoparticles on Au@Ag@void@infinite coordination polymers with oxidase-like activity for the degradation of methylene blue. (Reprinted with permission from [205]. Copyright © 2014, Royal Society of Chemistry).

Wang et al. [205] fabricated three-dimensional nano-assemblies of noble metal nanoparticle (NP)–infinite coordination polymers (ICPs) through the infiltration of HAuCl_4_ into hollow Au@Ag@ICPs core-shell nanostructures and its replacement reaction with Au@Ag nanoparticles (Figure 29). These 3D nano-assemblies possess specific oxidase-like activity. TMB was oxidised to generate its blue product using surface-adsorbed O_2_ on the surface without using H_2_O_2_. Methylene blue was also degraded using the oxidase-like activity of the 3D assemblies.

**Figure 30:**
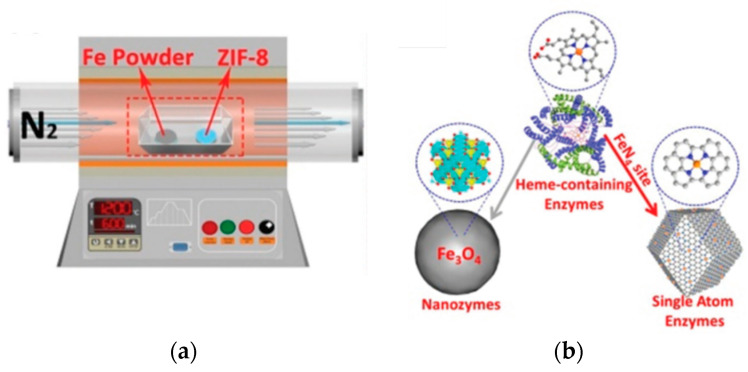
(**a**) Schematic illustration of the synthesis of iron single-atom nanozymes and (**b**) macrostructures and active sites of natural enzymes, nanozymes and iron single-atom nanozymes. (Reprinted with permission from [206]. Copyright © 2019, Royal Society of Chemistry).

Porphyrin-like single Fe sites on N-doped carbon nanomaterials (iron single-atom nanozymes, FeN_4_) were constructed by Zhao and co-workers [206] using highly specialised high-temperature techniques (Figure 30). These iron single-atom nanozymes exhibited excellent peroxidase-, oxidase- and catalase-like activities. The catalytic activities could be up 40 times higher than those of Fe_3_O_4_. The enhanced reactivity could be attributed to the FeN_4_ sites, which are similar to the natural heme-containing enzymes, the high surface area of the MOF (ZIF-8) structure and the large pore diameter (0.45 nm). These last two factors are beneficial for the mass transfer of reactant and product. TMB and OPD were the two substrates used for their colorimetric investigation, which showed promising results. The material was effective in the degradation of phenol in aqueous solution.

### 5.6. Potential Applications of Nanozymes in the Treatment of PFAS (Per- and Polyfluoroalkyl Substances) as Emerging Pollutants

Per- and polyfluoroalkyl substances, PFAS, are a large group of chemicals which have been used extensively in many commercial products including fire-fighting foams, lubricants, coatings, etc. These chemicals can travel great distances in the environment and have been known to be bioaccumulative. Although the toxicity of these compounds has not been fully understood, they are suspected to be the cause of carcinogenesis, mutagenesis and reproductive problems. They are now considered as emerging pollutants [207,208]. A number of technologies have emerged for the remediation of PFAS pollutants; however, the high stability of C-F bonds presents a great challenge in devising an effective method for the degradation of PFAS under mild conditions [207,208,209]. Recent works have shown that microbial and enzymes could affect the degradation of certain PFAS compounds. HRP successfully catalysed the degradation of perfluorooctanoic acid [210] and laccases could also catalyse the degradation of perfluorooctanoic acid [211]. Given the fact that many nanozymes have been demonstrated to have peroxidase and laccase activities as well have significant advantages over natural enzymes, nanozymes could play an important role in PFAS pollutant remediation efforts.

### 5.7. Summary

The peroxidase-like catalytic activity has been the activity most explored for the degradation of environmental pollutants such as phenols, Rh B, dyes (methylene blue, acid orange), etc. Nanozymes have several advantages including low cost, ease of preparation, high stability and recyclability. Zero valent metal-based nanozymes have been least utilised as catalysts for environmental pollutant remediation. Noble metals are expensive for large-scale applications and zero valent non-noble metals tend to be highly prone to oxidation and degradation. Moreover, 3D-structured materials that possess high specificity (moulded in active sites) have great potential in disease diagnosis. However, in pollutant degradation, high selectivity and specificity is often not required. Additionally, the manufacturing of 3D structures still requires significant technical efforts.

Composite nanozymes, particularly those with Fe_3_O_4_ MNPs nanoparticles on carbon materials or MOFs, have been shown to exhibit higher catalytic efficiency than metal-/metal-oxide-based nanozymes. One of the attractive features of these nanocomposites is that they have dual adsorption and degradation activities. The adsorptive ability of the base material, i.e., carbon or MOF, could bring the pollutant to the proximity of the active sites and increase the degradation efficiency of the composite nanozyme.

Most nanozymes were only efficient in the degradation of environmental pollutants under acidic pH conditions. This means that in wastewater treatment application, the treated wastewater will need to undergo an additional and potentially costly neutralisation step. Finding nanozymes that can degrade pollutants under neutral conditions would remarkably boost the application of nanozymes in this area.

## 6. Conclusions and Future Perspectives

Nanozymes have gathered increasing research interest since their first discovery less than 15 years ago [6] because they exhibit catalytic properties and offer improved tolerance to harsh conditions compared to natural enzymes. They have demonstrated applications in the biomedical and environmental fields, including diagnostics and therapeutics, sensing, environmental monitoring and remediation of environmental pollutants. In this review, we detailed the classification of nanozymes, their general catalytic mechanisms, as well as their recent progress in environmental applications through discriminating their diverse detection and remediation platforms.

Public concerns about environmental safety call for innovative and informative analytical techniques to meet the (i) detection requirements of high sensitivity and specificity, and (ii) remediation requirements of degradation efficiency to transform pollutants into another form less toxic to the environment. Nanozymes have emerged as an excellent tool to address both environmental detection and remediation requirements. Recent works have shown that nanozymes have fascinating catalytic properties, with added advantages over natural enzymes for environmental applications. Not only do they have wider operational windows (resistance to harsh environments), higher stability (long shelf life) and better recyclability, but they also offer tuneable surface functionality. The highly dynamic and active research has given rise to great opportunities in this field. It is believed that in certain applications, the combination of nanozymes with natural enzymes may lead to positive synergistic effects. However, an in-depth understanding of the fundamental principles of nanozymes for environmental quality and safety detection and remediation remains limited, which makes their applications largely empirical. To support sustainable growth and to realise the implementation of nanozymes in the environmental field, there is still scope for improvement.

1. Selectivity: Natural enzymes often have a defined size and morphology and catalyse a specific substrate or a class of analogues. However, nanozymes do not possess as high a substrate (target) selectivity as natural enzymes and their catalytic behaviours can be influenced by ions in the microenvironment, particularly anions. Consequently, more work in innovative surface engineering of nanozymes is still required to create target-tuneable catalysis which can perform selective recognition of the target.
2. Real-time in-field application: To meet the goal of real-time in-field detection of environmental pollutants, further studies are required to minimise sample pre-treatment (such as sample filtration, pH adjustment, solvent extraction, etc.). The vast majority of nanozymes for environmental pollutant monitoring and remediation are based on peroxidase-like activity, which has two drawbacks: (i) they require the addition of unstable hydrogen peroxide, and (ii) acidic pH adjustment to enhance signal. Ideally, nanozymes that can operate under neutral pH conditions without cumbersome sample modification can make the test more user-friendly. Furthermore, implementation of new and/or existing nanozymes into portable devices such as lateral flow assays and microfluidic chambers, coupled with the ability to capture data using portable optical readers such as smartphone cameras (for colorimetric detection), and combined with the fast data processing capability of a smartphone, will help to realise this goal.
3. Effectiveness and reusability of nanozymes: Nanocomposites between MNPs and carbon materials or MOFs have been shown to be remarkably more effective in the degradation of organic pollutants. The improved efficiencies are a result of the combined adsorption and catalytic abilities of the base materials and the MNPs and synergistic interactions between the two materials. Furthermore, while most nanozymes have optimal catalytic activity at acidic pH, examples of composite nanozymes which are highly active at neutral pH are also known. Accordingly, further studies into the development of nanozymes that are highly active at neutral pH will be beneficial for both the detection and the remediation of pollutants as this will reduce sample pre- and post-treatments, e.g., no acidification or post-neutralisation steps are required. Additionally, nanozymes that can be regenerated are also helpful, particularly in the remediation of environmental pollutants (such that they can continue to degrade more pollutants). Thus, reusability and ecological compatibility are also valuable considerations when designing new remediation strategies.
4. Commercial production: Currently, most nanoparticles used in research studies are synthesised in small batches using methods which are usually labour-intensive and time-consuming. Since nanoparticles have a large surface area to volume (and mass) ratio with greater reactivity and mobility, they have the tendency to agglomerate into larger microparticles, losing their distinctive nano characteristics. Small synthetic batches are also prone to batch-to-batch variability and a wide particle size distribution, which can significantly affect the yield of production. While small batches are adequate for early studies, this limits the translation of promising nanozymes for commercial deployment in environmental applications, where orders of magnitude more material are required. Development of methodologies such as flow chemistry techniques that can produce gram quantities per hour of highly reproducible nanozymes, combined with coating strategies to reduce agglomeration at the commercial scale, is important to support scale-up production.
5. Industrial standards and regulation: There is no doubt that nanozymes provide industries with many advantages. However, there are concerns related to the impact of nanoparticles on the ecological system, especially when deployed at large scale for remediation. Current regulations establish metal content limits without consideration of particle size. While implementing nanozymes as an environmental remediation tool, the development should be coupled with appropriate measurement techniques that can quantify both concentrations and particle sizes with appropriate quality assurance and quality control. As well as size and composition, it is evident that the surface properties of nanoparticles will be fundamental in determining the fate and toxicity in the environment and that these properties will need to be considered in any hazard ranking. Consequently, it is necessary to develop computational models to correlate the physicochemical properties of such nanozymes with their potential nanotoxicity. These models can also support public needs and industrial regulations for future remediation designs.

## Figures and Tables

**Table 1:** Examples of metalloenzymes.

Metal Centre	Enzymes
Zinc	Carbonic anhydrase, alcohol dehydrogenase, organophosphate hydrolase
Iron	Catalase, peroxidase, cytochrome oxidase
Manganese	Enolase, hexokinase
Copper	Tyrosinase, lysyl oxidase, laccase
Cobalt	Dipeptidase

**Table 4:** Examples of type II nanozymes in environmental pollutant remediation.

Nanozyme	Pollutant	Enzyme-Like Activity	Year	Ref.
Fe(0) nanoparticles	4-Chloro-3-methyl phenol	Peroxidase	2011	[183]
Carbon nanodots	Azo dyes (methyl red and methyl orange)	Peroxidase	2012	[182]
Cubic boron nitride	Rh B	Peroxidase	2016	[184]
Graphitic carbon nitride	TMB	Dual oxidase-peroxidase	2019	[185]

**Table 5:** Examples of type III nanozymes in environmental pollutant remediation.

Nanozyme	Pollutant	Enzyme-Like Activity	Year	Ref.
Fe_3_O_4_ MNP/MWNT composites	Phenol	Peroxidase	2009	[186]
Fe_2_(MoO_4_)_3_	Acid Orange II	Peroxidase	2011	[190]
ɣ-FeOOH/(reduced graphene oxide)	Phenol	Peroxidase	2011	[187]
Fe_3_O_4_/CeO_2_ nanocomposites	4-Chlorophenol	Peroxidase	2012	[158]
Fe_3_O_4_/MSU-F-C (magnetite-loaded mesocellular carbonaceous material)	Arsenic, phenol	Peroxidase	2012	[188]
Fe_x_O_y_-MWNT	Orange G	Peroxidase	2012	[199]
MIL-101 (chromium(III) terephthalate metal organic framework) (chelated to *N,N*-dimethylamino pyridine)	Paraoxon	Hydrolase	2013	[197]
Porous Co_3_O_4_ nanorods–reduced graphene oxide	Methylene blue	Peroxidase	2013	[191]
Fe_3_O_4_/reduced graphene oxide nanocomposites	Methylene blue	Peroxidase	2013	[200]
graphene oxide quantum dots/Fe_3_O_4_ composites	Phenolic compounds	Peroxidase	2014	[48]
graphene oxide/Fe_3_O_4_ nanocomposites	Acid Orange 7	Peroxidase	2014	[192]
Polyprrole/hemin (PPy/hemin) nanocomposite	Methyl orange and Rh B	Pollutant adsorbent (not enzymatic activity)	2014	[193]
Fe_3_O_3_/graphene oxide	Rh B	Peroxidase	2014	[201]
CeO_2_/ɣ-Fe_2_O_3_	Parathion-methyl, nerve agents soman and VX	Peroxidase	2015	[198]
Fe_3_O_4_/Al-B (Fe_3_O_4_ MNPs decorated Al pillared bentonite)	Rh B	Peroxidase + adsorption	2015	[194]
Fe_3_O_4_/CNTs	Orange II	Peroxidase	2016	[195]
C-CoM-HNCs (HNC = homobimetallic hollow cages), (C-CoM-HNC M = Ni, Mn, Cu and Zn)	Rh B	Oxidase	2019	[196]
DhH6-*c*-ZrMOF (deterohemin-peptide on Zr metal organic framework)	Phenol	Peroxidase	2020	[189]
Cellulose incorporated magnetic nano-biocomposites, Fe_3_O_4_ on cellulose	Methyl orange, textile effluent	Peroxidase	2020	[159]

**Table 6:** Examples of type IV nanozymes in environmental pollutant remediation.

Nanozyme	Pollutant	Enzyme-Like Activity	Year	Ref.
Porous Fe_3_O_4_ nanospheres	Xylenol Orange	Peroxidase	2011	[204]
3D nano-assembly of Au nanoparticles on Au@Ag@ICPs	TMB and methylene blue	Oxidase	2015	[205]
Iron single-atom nanozyme, FeN_4_	TMB and OPD	Peroxidase, oxidase and catalase	2019	[206]

## Data Availability

Data sharing not applicable.

## Glossary

AAS	atomic absorption spectroscopy
ABTS	2,2′-azino-bis-(3-ethylbenzothiazoline-6-sulfonate)
AChE	acetylcholinesterase
BPA	bisphenol A
CHO	choline oxidase
EDTA	ethylenediaminetetraacetic acid
GC-MS	gas chromatography–mass spectrometry
HRP	horseradish peroxidase
ICP-MS	inductively coupled plasma–mass spectrometry
ICP-OES	inductively coupled plasma–optical emission spectroscopy
LC-MS	liquid chromatography–mass spectrometry
MNP	magnetic nanoparticles
MOF	metal–organic framework
MWNT	multiwalled carbon nanotube
OPD	*o*-phenylenediamine
PFAS	per- and polyfluoroalkyl substances
Rh B	Rhodamine B
TMB	3,3′,5,5′-tetramethylbenzidine
